# Differential effects of anti-CD20 therapy on CD4 and CD8 T cells and implication of CD20-expressing CD8 T cells in MS disease activity

**DOI:** 10.1073/pnas.2207291120

**Published:** 2023-01-12

**Authors:** Koji Shinoda, Rui Li, Ayman Rezk, Ina Mexhitaj, Kristina R. Patterson, Mihir Kakara, Leah Zuroff, Jeffrey L. Bennett, H.-Christian von Büdingen, Robert Carruthers, Keith R. Edwards, Robert Fallis, Paul S. Giacomini, Benjamin M. Greenberg, David A. Hafler, Carolina Ionete, Ulrike W. Kaunzner, Christopher B. Lock, Erin E. Longbrake, Gabriel Pardo, Fredrik Piehl, Martin S. Weber, Tjalf Ziemssen, Dina Jacobs, Jeffrey M. Gelfand, Anne H. Cross, Briana Cameron, Bruno Musch, Ryan C. Winger, Xiaoming Jia, Christopher T. Harp, Ann Herman, Amit Bar-Or

**Affiliations:** ^a^Department of Neurology, Perelman School of Medicine, University of Pennsylvania, Philadelphia, PA 19104; ^b^Center for Neuroinflammation and Experimental Therapeutics, Perelman School of Medicine, University of Pennsylvania, Philadelphia, PA 19104; ^c^Departments of Neurology and Ophthalmology, Programs in Neuroscience and Immunology, University of Colorado School of Medicine, Aurora, CO 80045; ^d^F. Hoffmann-La Roche, 4070 Basel, Switzerland; ^e^Department of Medicine, University of British Columbia, Vancouver, BC V6T 2B5, Canada; ^f^Multiple Sclerosis Center of Northeastern New York, Comprehensive MS Care Center Affiliated with the National MS Society, Latham, NY 12110; ^g^Department of Neurology, Ohio State University Medical Center, Columbus, OH 43210; ^h^Department of Neurology and Neurosurgery, Montreal Neurological Institute, McGill University, Montreal, QC H3A 2B4, Canada; ^i^Department of Neurology, University of Texas Southwestern Medical Center, Dallas, TX 75390; ^j^Departments of Neurology and Immunobiology, Yale School of Medicine, New Haven, CT 06510; ^k^Department of Neurology, University of Massachusetts Medical School, Worcester, MA 01655; ^l^Judith Jaffe Multiple Sclerosis Center, Weill Cornell Medicine, New York, NY 10021; ^m^Department of Neurology and Neurological Sciences, Stanford University, Palo Alto, CA 94304; ^n^Department of Neurology, Yale University, New Haven, CT 06510; ^o^Oklahoma Medical Research Foundation, Multiple Sclerosis Center of Excellence, Oklahoma City, OK 73104; ^p^Department of Clinical Neuroscience, Karolinska Institute, SE-171 76 Stockholm, Sweden; ^q^Department of Neurology, Karolinska University Hospital, SE-171 77 Stockholm, Sweden; ^r^Neuroimmunology Unit, Center for Molecular Medicine, Karolinska University Hospital, Karolinska Institute, SE-171 77 Stockholm, Sweden; ^s^Institute of Neuropathology, University Medical Center, 37075 Göttingen, Germany; ^t^Department of Neurology, University Medical Center, 37075 Göttingen, Germany; ^u^Fraunhofer-Institute for Translational Medicine and Pharmackology ITMP, 37075 Göttingen, Germany; ^v^Department of Neurology, Center of Clinical Neuroscience, University Hospital Carl Gustav Carus, Technical University of Dresden, 01307 Dresden, Germany; ^w^Weill Institute for Neurosciences, University of California, San Francisco, CA 94158; ^x^Department of Neurology, University of California, San Francisco, CA 94158; ^y^Department of Neurology, Washington University School of Medicine, Saint Louis, MO 63110; ^z^Genentech, Inc., South San Francisco, CA 94080; ^aa^Children's Hospital of Philadelphia, University of Pennsylvania, Philadelphia, PA 19104

**Keywords:** anti-CD20 therapy, ocrelizumab, CD20-expressing T cells, CD20^dim^ T cells, CD20^dim^CD8^+^ T cells

## Abstract

A small proportion of multiple sclerosis (MS) patients develop new disease activity soon after starting anti-CD20 therapy. This activity does not recur with further dosing, possibly reflecting deeper depletion of CD20-expressing cells with repeat infusions. We assessed cellular immune profiles and their association with transient disease activity following anti-CD20 initiation as a window into relapsing disease biology. Peripheral blood mononuclear cells from independent discovery and validation cohorts of MS patients initiating ocrelizumab were assessed for phenotypic and functional profiles using multiparametric flow cytometry. Pretreatment CD20-expressing T cells, especially CD20^dim^CD8^+^ T cells with a highly inflammatory and central nervous system (CNS)-homing phenotype, were significantly inversely correlated with pretreatment MRI gadolinium-lesion counts, and also predictive of early disease activity observed after anti-CD20 initiation. Direct removal of pretreatment proinflammatory CD20^dim^CD8^+^ T cells had a greater contribution to treatment-associated changes in the CD8^+^ T cell pool than was the case for CD4^+^ T cells. Early disease activity following anti-CD20 initiation was not associated with reconstituting CD20^dim^CD8^+^ T cells, which were less proinflammatory compared with pretreatment. Similarly, this disease activity did not correlate with early reconstituting B cells, which were predominantly transitional CD19^+^CD24^high^CD38^high^ with a more anti-inflammatory profile. We provide insights into the mode-of-action of anti-CD20 and highlight a potential role for CD20^dim^CD8^+^ T cells in MS relapse biology; their strong inverse correlation with both pretreatment and early posttreatment disease activity suggests that CD20-expressing CD8^+^ T cells leaving the circulation (possibly to the CNS) play a particularly early role in the immune cascades involved in relapse development.

## Significance

CD20-expressing CD4^+ ^and CD8^+ ^T cells harbor proinflammatory and central nervous system (CNS)-homing attributes. The inverse relationship between levels of these cells (particularly the CD20^dim^CD8^+ ^T cells) in the circulation of MS patients, with active and impending CNS inflammation, suggests that these cells participate early on in the cellular immune responses involved in relapse development. The differential effects of anti-CD20 treatment on CD4^+ ^and CD8^+ ^T cell subsets point to different contributions of direct removal of CD20-expressing T cells, as well as indirect effects likely reflecting the removal of B cells that alters in vivo T cell:B cell interactions.

While anti-CD20 (aCD20) therapy has proven highly successful in the treatment of multiple sclerosis (MS) and is approved for use in patients with both relapsing–remitting MS (RRMS) and primary progressive MS (PPMS) (1–8), concepts surrounding its therapeutic mode of action in MS have continued to evolve. The original studies of aCD20 in patients with MS were pursued with the view that depletion of CD20-expressing B cells may be beneficial given the long-standing recognition of abnormally elevated cerebrospinal fluid (CSF) immunoglobulins and demonstration of antibodies bound to myelin within phagocytic cells of MS lesions, as well as the presence of oligoclonal bands and the clonal persistence of intrathecal B cells (9, 10). However, studies showing no major treatment effects of aCD20 on the abnormal CSF antibody profiles of patients (11–13) at the same time that they benefit from much-reduced new disease activity, shifted attention to antibody-independent contributions of B cells to the development of new MS relapses. A growing body of work ensued, highlighting the presence and ability of abnormally proinflammatory B cells of untreated MS patients to serve as antigen-presenting cells and/or secrete proinflammatory cytokines, capable of aberrantly activating potentially pathogenic T cells and myeloid cells (14–25). More recently, the realization that small subsets of (both CD4^+^ and CD8^+^) T cells can also express low levels of CD20 (referred to as CD20^dim^), and that these cells are also depleted with aCD20 (26–30), has raised an alternate (though not mutually exclusive) possibility, that the ability of aCD20 agents to limit relapsing MS disease activity may in part be mediated by direct removal of pathogenic T cells expressing CD20. The demonstration that removal of CD20^+^ T cells ameliorated disease in the experimental autoimmune encephalomyelitis animal model has provided proof-of-principle that CD20-expressing T cells can be involved in central nervous system (CNS) inflammation (31), though evidence for such involvement in the human disease is lacking.

Of note, in spite of being highly effective at limiting relapsing MS disease activity, a small proportion of patients nonetheless exhibit new disease activity after starting aCD20 treatment—typically within the first 3 to 6 mo following treatment initiation (5, 6). The mechanism underlying this disease activity has not been formally defined, and we postulated that examining the cellular immune profiles of patients prior to and following aCD20 initiation, and relating these to measures of disease activity, could help elucidate contributions of particular immune-cell subsets to MS relapse pathophysiology. To this end, we studied two well-characterized (discovery and validation) cohorts of MS patients initiating aCD20 (ocrelizumab) treatment to first define phenotypic and functional profiles of circulating subsets of both T cells and B cells—prior to and during depletion and early reconstitution phases. We then assessed the association of particular immune-cell subsets (at “baseline” and during treatment), with the presence and development of MS disease activity (defined based on clinical and/or MRI measures). Our findings help elucidate mechanisms underlying this early disease activity, implicating a particular cell subset with insights into the timing when these cells may be involved in the cascades of immune-cell interactions that contribute to relapsing MS disease activity.

## Results

### Study Participants.

Immune phenotyping was performed in samples from two independent (discovery and validation) cohorts of MS patients initiating treatment with ocrelizumab (Table 1). Participants in the “discovery cohort” (*SI Appendix*, Table S1 for additional details) included 23 MS patients with either RRMS or PPMS with no prior exposure to disease-modifying therapy (DMT), who were enrolled at a single academic center (U Penn), and provided blood samples prior to initiation of ocrelizumab (pretreatment) and again between 2 and 4 mo after treatment-initiation. The validation cohort (*SI Appendix*, Table S2) focused on RRMS, and comprised of 35 patients enrolled in a formal open-label, multicenter, biomarker study (https://clinicaltrials.gov/ct2/show/NCT02688985), who underwent blood sampling as well as standardized clinical assessments and coregistered research brain MRI scans pretreatment and then at weeks 12, 24, and 52, in order to capture both clinically evident as well as subclinical new disease activity. Among the 35 patients in the validation cohort, 19 (54.3%) had no prior DMT exposure, while the others had prior exposure to glatiramer acetate (n = 7), interferon (IFN)-β (n = 5), dimethyl fumarate (n = 3), or fingolimod (n = 1).

**Table 1.** Basic demographics of discovery and validation cohorts

Demographics	Discovery cohort(n = 23)	Validation cohort(n = 35)
Age, mean (sd)	48.2 (13.3)	37.3 (10.3)
Female gender, n (%)	12 (52.2%)	21 (60.0%)
Diagnosis, n (%)		
RRMS	13 (56.5%)	35 (100%)
SPMS or PPMS	10 (43.5%)	–
Race, n (%)		
White	13 (56.5%)	29 (82.9%)
Black/AfricanAmerican	7 (30.4%)	5 (14.3%)
Asian	0 (0%)	1 (2.9%)
Others/unknown	3 (13.0%)	0 (0%)
Disease duration in years; mean (sd)^*^	5.3 (5.2)	6.8 (8.9)
Duration from last relapse in months; mean (sd)^†^	11.5 (11.9)	15.6 (8.8)
EDSS score at baseline, mean (sd)	2.7 (1.8)	2.1 (1.2)
Prior DMT treatment, n (%)		
Treatment-naive	23 (100%)	19 (54.3%)
Glatiramer acetate	–	7 (20.0%)
Interferon-β	–	5 (14.3%)
Dimethyl fumarate	–	3 (8.6%)
Fingolimod	–	1 (2.9%)

Discovery cohort participants were recruited at a single academic center (U Penn) while the independent validation cohort participants were recruited as part of a formal open-label, multicenter, biomarker study (https://clinicaltrials.gov/ct2/show/NCT02688985).

^*^Disease duration from symptom onset.

^†^Data available for all 13 patients with relapsing MS in the discovery cohort and 31 of 35 patients in the validation cohort. Abbreviations: DMT, disease-modifying therapy; EDSS, Expanded Disability Status Scale; PBMC, peripheral blood mononuclear cells; MS, multiple sclerosis; RRMS, relapsing–remitting MS; PPMS, primary progressive MS; sd, standard deviation.

### The Impact of aCD20 Initiation on Immune-Cell Subsets.

**Fig. 1.**
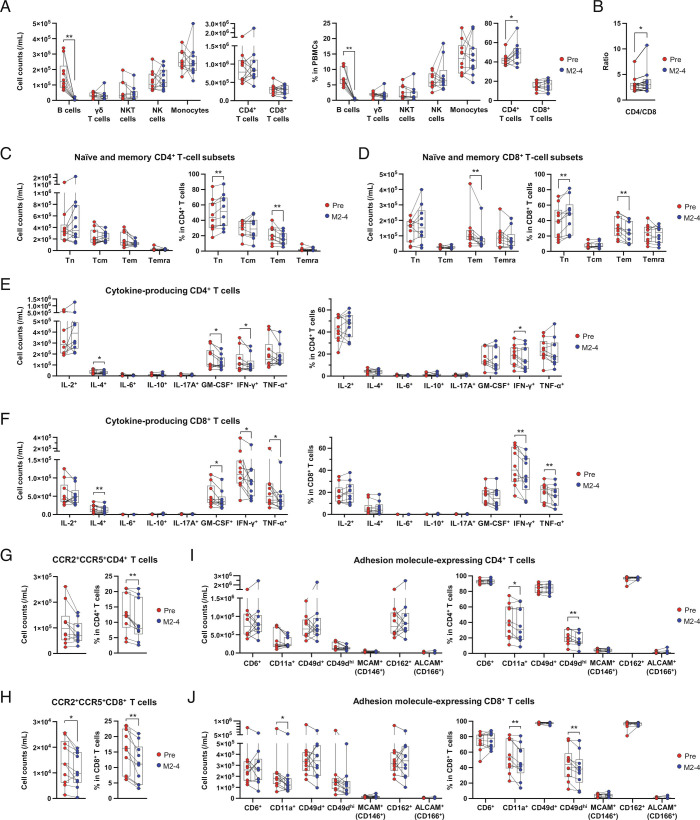
aCD20 treatment alters CD4^+^ and CD8^+^ T cell phenotype—discovery cohort. Changes in immune-cell subsets of 10 MS patients between pretreatment (Pre) and 2 to 4 mo (M2–4) after aCD20 (ocrelizumab) initiation (see *SI Appendix, *Fig. S1 for flow cytometry gating strategy). (*A*) Absolute counts and frequencies of major immune-cell subsets including CD19^+^ B cells, CD4^+^ T cells, CD8^+^ T cells, γδ T cells, NKT, and NK cells. (*B*) Effect of ocrelizumab on the CD4^+^/CD8^+^ T cell ratio. (*C* and *D*) Absolute counts and frequencies of major T cell subsets, including naive (Tn, CCR7^+^CD45RA^+^), central memory (Tcm, CCR7^+^CD45RA^−^), effector memory (Tem, CCR7^−^CD45RA^−^) and terminally differentiated effector memory (Temra, CCR7^−^CD45RA^+^) among CD4^+^ T cells (*C*), and among CD8^+^ T cells (*D*). (*E* and *F*) Absolute counts and frequencies of cytokine-producing CD4^+^ (*E*) and CD8^+^ T cells (*F*) after ex vivo stimulation with phorbol 12-myristate 13-acetate and ionomycin for 4 h. (*G* and *H*) Absolute counts and frequencies of CCR2^+^CCR5^+^CD4^+^ (*G*), and CCR2^+^ CCR5^+^ CD8^+^ T cells (*H*). Absolute counts and frequencies of adhesion molecule-expressing CD4^+^ T cells (*I*) and CD8^+^ T cells (*J*). Statistical analysis was performed using the Wilcoxon matched-pairs signed-rank test followed by multiple comparison correction using the false discovery rate by Benjamini, Krieger, and Yekutieli's two-stage step-up method. *P* value, *P < 0.05, **P < 0.01, ***P < 0.001, ****P < 0.0001.

The impact of initiating aCD20 treatment (ocrelizumab) on circulating immune cells in the discovery cohort of (previously DMT-naïve) MS patients was assessed by flow cytometry (*SI Appendix*, Fig. S1) with results shown in Fig. 1. Absolute counts of major immune-cell types (Fig. 1*A*), were not appreciably impacted, with the exception of the expected substantial (>95%) decreases in absolute counts and frequencies of circulating B cells. There appeared to be a small increase in the CD4/CD8 T cell ratio (*P* = 0.019) (Fig. 1*B*). When assessing subsets of T cells, the frequency of CD4^+^ effector memory (Tem) T cells was modestly reduced (by 22.0%, *P* = 0.002) with a relative increase in the frequency of CD4^+^ naive (Tn) T cells (*P* = 0.002) following ocrelizumab initiation (Fig. 1 *C* and *D*). Similar changes were observed for corresponding CD8^+^ T cell subsets, with aCD20 treatment appearing to result in decreases in both absolute counts (by 29.1%, *P* = 0.018) and frequencies (*P* = 0.004) of CD8^+^ Tem cells, and an increase in the frequency of CD8^+^ naïve (Tn) cells (*P* = 0.004) (Fig. 1 *C* and *D*). In keeping with decreases in the more differentiated Tem subset, CD8^+^ T cells expressing differentiation and exhaustion markers, including inhibitory receptors such as programmed death (PD)-1, 2B4, T cell immunoglobulin with immunoreceptor tyrosine-based inhibitory motif domain (TIGIT), and Eomes-expressing nonnaive CD8^+^ T cells, were decreased in both cell number and frequency (*SI Appendix*, Fig. S2) following aCD20 treatment initiation.

These initial observations suggested that aCD20 treatment resulted in small decreases in the circulation of the more differentiated (rather than naïve) CD4^+^ and CD8^+^ T cell subsets. To further explore which functional subsets of differentiated T cells may be affected, we considered subsets previously implicated in MS pathophysiology, including proinflammatory cytokine-expressing T cells as well as T cells expressing chemokine receptors and adhesion molecules known to be involved in CNS trafficking (32–38). Ex vivo stimulation and intracellular cytokine staining revealed decreased numbers and frequencies of multiple circulating cytokine-defined T cell subsets following aCD20 treatment (Fig. 1 *E* and *F*). This included decreases in circulating proinflammatory (granulocyte-macrophage colony-stimulating factor (GM-CSF) and IFN-γ-expressing) CD4^+^ T cells and proinflammatory (GM-CSF, IFN-γ, and tumor necrosis factor (TNF)-α-expressing) CD8^+^ T cells. Also decreased were both counts and frequencies of CNS-homing CCR2^+^CCR5^+^CD4^+^ and CCR2^+^CCR5^+^CD8^+^ T cells, as well as frequencies of CD4^+^ and CD8^+^ T cells expressing CD11a (integrin αL) which is the α chain of the αLβ2 integrin lymphocyte function-associated antigen 1, or CD49d (integrin α4) which is the α chain of very late antigen-4 (VLA4) (Fig. 1 *G–J*). In contrast to the decreases in Th1, Tc1-like and CNS-homing proinflammatory CD4^+^ and CD8^+^ T cells, we found no decreases in counts or frequencies of interleukin (IL)-10-expressing or phenotypically defined (CD25^+^CD127^lo/−^) regulatory CD4^+^ T cells (Tregs), their subsets, or their expression levels of Treg-associated molecules (Foxp3, CD39, TIGIT; *SI Appendix*, Fig. S3).

To confirm key observations from the discovery cohort, we evaluated the impact of ocrelizumab initiation in an independent, prospectively followed cohort, focusing on patients with RRMS (*SI Appendix*, Table S2 and Fig. S4). The observation of a small but statistically significant increase in the CD4/CD8 T cell ratio following ocrelizumab initiation was replicated and appeared to persist until just prior to the next ocrelizumab infusion at week 24 (W24). Decreases in both CD4^+^ and CD8^+^ Tem cell counts (22.4% and 39.1% reductions, respectively) and frequencies were also confirmed following ocrelizumab initiation, and tended to persist through W24. In keeping with this, the observations that aCD20 treatment results in decreased counts of circulating proinflammatory (GM-CSF^+^ and IFN-γ^+^) CD4^+^ T cells and proinflammatory (GM-CSF^+^, IFN-γ^+^, and TNF-α^+^) CD8^+^ T cells, as well as CNS-homing and activated CCR2^+^CCR5^+^ and CD11a^+^ CD4^+^ and CD8^+^ T cells, were also confirmed.

### Assessing Whether the Impact of aCD20 Treatment on T Cell Subsets May Be Attributed to Direct Removal of CD20-Expressing T Cells.

Small populations of CD4^+^ and CD8^+^ T cells are known to express low levels of CD20 (referred to as CD20^dim^ T cells). These CD20^dim^ T cells, which are also depleted with aCD20 therapies, have been described as proinflammatory (26, 27, 29, 30). We therefore wished to assess the extent to which the effects of aCD20 that we observed on circulating proinflammatory CD4^+^ and CD8^+^ T cell subsets could be explained by direct removal of the CD20^dim^ subsets of T cells. However, the use of aCD20 antibodies to investigate circulating cells in patients treated with aCD20 has been controversial because of concerns over the potential masking of surface CD20 by still-circulating aCD20 antibody, which could result in false-negative assessment since actual CD20-expressing cells may not be detected in spite of being present (39). To address this, we combined intracellular and surface staining for CD20 in peripheral blood mononuclear cell (PBMC) samples from a number of MS patients prior to and following aCD20 treatment (*SI Appendix*, Fig. S5). Using two distinct aCD20 antibody clones, one of which (clone 2H7) binds to the same extracellular epitope of CD20 as ocrelizumab, and another (clone 1412), which binds to a distinct intracellular epitope of CD20, we found that the substantial loss of T cell staining for surface CD20 following aCD20 treatment corresponded to the loss of the T cell staining for cytoplasmic CD20, indicating these cells were indeed depleted rather than being masked.

**Fig. 2.**
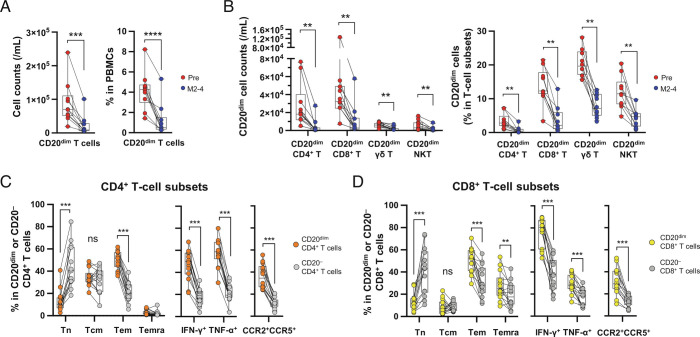
Efficient depletion by aCD20 treatment of CD20^dim^ T cells that are highly enriched for proinflammatory effector cells. (*A*) Absolute cell counts and frequencies of CD20^dim^ T cells of 10 MS patients at baseline (Pre) and months 2 to 4 (M2–4) after the initiation of ocrelizumab. (*B*) Cell counts and frequencies of CD20^dim^ cells within CD4^+^ T cells, CD8^+^ T cells, γδ T cells, and NKT cells. (*C*) Comparison of surface or intracellular markers between CD20^dim^CD4^+^ and CD20^−^CD4^+^ cells at baseline (n = 14). (*D*) Comparison of surface or intracellular markers between CD20^dim^CD8^+^ and CD20^−^CD8^+^ cells at baseline (n = 14). Abbreviations: ns, not significant; Tcm, central memory T cells; Tem, effector memory T cells; Temra, terminally differentiated effector memory T cells; Tn, naive T cells. Statistical analysis was performed using the Wilcoxon matched-pairs signed-rank test (*A* and *B*) and Wilcoxon matched-pairs signed-rank test followed by multiple comparison correction using the false discovery rate by Benjamini, Krieger, and Yekutieli's two-stage step-up method (*C* and *D*). *P* value, *P < 0.05, **P < 0.01, ***P < 0.001, ****P < 0.0001.

We were then able to interrogate CD20-expressing cells both prior to and following aCD20 treatment (Fig. 2 *A* and *B*). Consistent with prior reports (27, 29, 30), we observed that in pretreatment (Pre) samples of our discovery cohort, CD20 was expressed by a small subset (3.1%) of CD4^+^ T cells and a somewhat larger subset (13.4%) of CD8^+^ T cells (Fig. 2*B*). We further identified populations of γδ T cells and natural killer T (NKT) cells expressing CD20 (Fig. 2*B*), also present at higher frequencies than the CD20-expressing CD4^+^ T cells (Fig. 2*B*). aCD20 treatment resulted in substantial decreases in absolute counts and frequencies of total CD3^+^ T cells expressing CD20 (Fig. 2*A*) as well as in all CD20-expressing T cell subsets (Fig. 2*B*). The profile and impact of aCD20 on all of these CD20-expressing T cells was confirmed in the validation cohort (*SI Appendix*, Fig. S6 *A* and *B*).

**Fig. 3.**
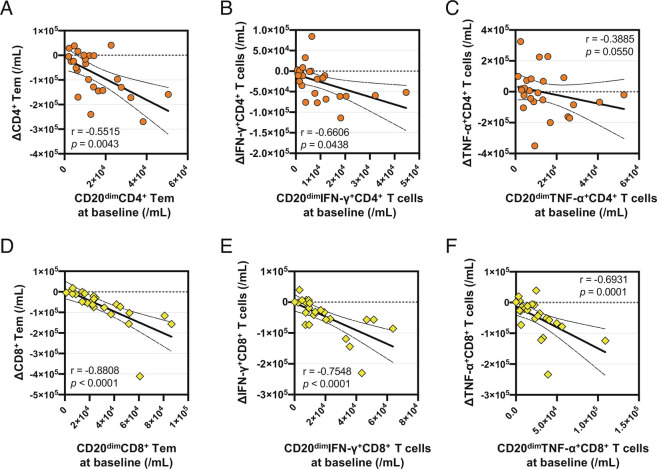
Correlations between treatment-induced changes in CD4^+^ and CD8^+^ T cell counts, and pretreatment (baseline) counts of CD20^dim^CD4^+^ and CD8^+^ T cell subsets. Correlations between: (*A*) treatment-induced changes (Δ) in CD4^+^ Tem counts and pretreatment (baseline) counts of CD20^dim^CD4^+^ Tem cells; (*B*) treatment-induced changes in IFN-γ^+^CD4^+^ T cell counts and pretreatment counts of CD20^dim^IFN-γ^+^CD4^+^ T cells; (*C*) treatment-induced changes in TNF-α^+^CD4^+^ T cell counts and pretreatment counts of CD20^dim^TNF-α^+^CD4^+^ T cells; (*D*) treatment-induced changes in CD8^+^ Tem counts and pretreatment counts of CD20^dim^CD8^+^ Tem; (*E*) treatment-induced changes in IFN-γ^+^CD8^+^ T cell counts and pretreatment counts of CD20^dim^IFN-γ^+^CD8^+^ T cells; (*F*) treatment-induced changes in TNF-α^+^CD8^+^ T cell counts and pretreatment counts of CD20^dim^TNF-α^+^CD8^+^ T cells. Statistical analysis was performed using Spearman correlation coefficient.

We considered the extent to which removal of the CD20-expressing T cell subsets that were present pretreatment in individual patients would account for changes seen in the CD4^+^ and CD8^+^ T cell compartments of the same patient following aCD20 treatment. To approach this, we first comprehensively characterized the pretreatment phenotypic and functional profiles of CD20-expressing CD4^+^ and CD8^+^ T cells, which would enable us to subsequently assess the effects of aCD20 treatment on T cells with corresponding profiles. Unlike CD20-negative T cells, the pretreatment CD20-expressing CD4^+^ T cells (Fig. 2*C*) and CD8^+^ T cells (Fig. 2*D*) were highly enriched with Tem but not Tn cells, harbored high proportions of proinflammatory cytokine-expressing cells, and expressed significantly higher levels of CNS-homing chemokine receptors (Fig. 2 and *SI Appendix*, Figs. S6 *C* and *D* and S7). We next postulated that if removal of pretreatment CD20-expressing T cells contributed to changes seen in T cell subsets following aCD20 treatment, there would be a strong correlation between treatment-induced changes in counts of CD4^+^ and CD8^+^ T cell subsets, and the pretreatment counts of corresponding CD20-expressing T cells. Pooled analysis of the combined discovery and validation cohorts (Fig. 3) revealed particularly strong correlations for CD8^+^ Tem (Fig. 3*D*) and proinflammatory CD8^+^ T cell subsets including IFN-γ-expressing (Fig. 3*E*) and TNF-α-expressing (Fig. 3*F*) CD8^+^ T cells. While similar trends were seen for CD4^+^ Tem and the proinflammatory CD4^+^ T cell subsets, these correlations tended to be weaker (Fig. 3 *A*–*C*). For example, while the removal of pretreatment CD20-expressing CD8^+^ Tem cells accounted on average for 38% of the decrease in total Tem cells, reductions of pretreatment CD20-expressing CD4^+^ Tem cells accounted for only 11% of the decrease in total Tem cells. A similar pattern of correlations was noted when comparing treatment-induced changes in counts of CD4^+^ and CD8^+^ T cell subsets, and treatment-induced changes of corresponding CD20-expressing T cells (*SI Appendix*, Fig. S8). Overall, these findings suggest that direct removal of pretreatment proinflammatory CD20-expressing CD8^+^ T cells had a greater contribution to treatment-associated changes in the CD8^+^ T cell pool than was the case for CD4^+^ T cells.

### Immune-Cell Associations with MS Disease Activity prior to and Following Initiation of aCD20 Treatment.

**Fig. 4.**
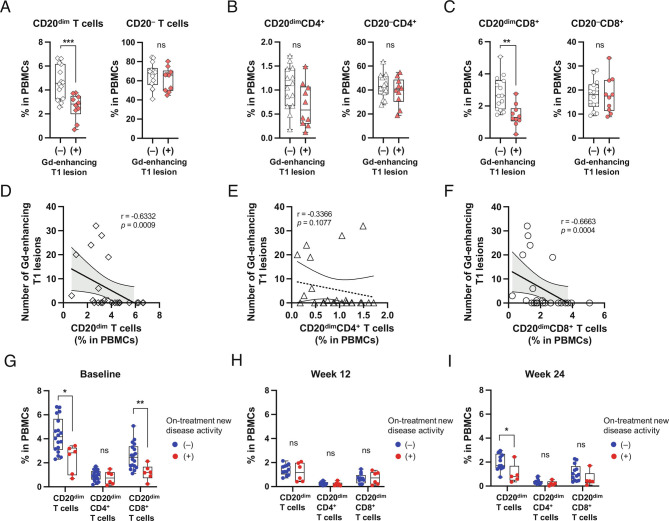
MS disease activity is associated with decreases in CD20-expressing T cells. (*A*–*C*) Comparison of the percentages of (*A*) CD20^dim^ and CD20^–^ T cells; (*B*) CD20^dim^ and CD20^−^CD4^+^ T cells; and (*C*) CD20^dim^ and CD20^−^CD8^+^ T cells (*C*) at baseline between MS patients with and without Gadolinium (Gd)-enhancing T1 lesion at baseline (n = 14, n = 10, respectively). (*D*–*F*) Correlation between the number of Gd-enhancing T1 lesion at baseline and the frequency of CD20^dim^ T cells (*D*); CD20^dim^CD4^+^ T cells (*E*); and CD20^dim^CD8^+^ T cells (*F*). Frequencies of CD20-expressing T cells assessed at baseline (*G*); and at week 12 (*H*); and week 24 (*I*) after ocrelizumab treatment initiation, were compared between patients who did (red symbols) or did not (blue symbols) experience new disease activity beyond 12 wk of ocrelizumab treatment (see also *SI Appendix, *Table S3). Statistical analysis was performed using the unpaired *t* test, Mann–Whitney test, or Spearman correlation coefficient. *P* value, *P < 0.05, **P < 0.01, ***P < 0.001.

Inclusion of clinical assessments and standardized serial MRIs in the validation cohort enabled us to assess the relationship between new MS disease activity (monitored both clinically and subclinically) and particular immune-cell subsets, or changes in immune-cell subsets following treatment initiation. We first considered whether particular pretreatment cell subsets were associated with disease activity present at that time. Among 24 patients with available Gadolinium (Gd)-infused scans at baseline, 10 (42%) had at least one Gd+ brain lesion. A striking observation was that the frequency of circulating CD20-expressing T cells was significantly lower in patients with Gd+ lesions compared with those without Gd+ lesions, which was not the case for the CD20-negative T cells (Fig. 4*A*). This was particularly evident for the CD20^dim^CD8^+^ T cells (Fig. 4*C*), and to a lesser extent for CD20^dim^CD4^+^ T cells (Fig. 4*B*). Indeed, the number of Gd+ lesions pretreatment correlated inversely with the frequency of total circulating CD20^dim^ T cells in the same patients (Fig. 4*D*), driven by a strong correlation with the CD20^dim^CD8^+^ T cells (Fig. 4*F*), which was not seen for the CD20^dim^CD4^+^ T cells (Fig. 4*E*). No other pretreatment immune-cell subsets (whether B cells, total CD4^+^ T cells, total CD8^+^ T cells, or natural killer (NK) cells) were associated with disease activity (*SI Appendix*, Fig. S9). Thus, pretreatment, the most striking association between immune-cell profiles and MS disease activity was an inverse correlation between CD20-expressing CD8^+^ T cells and the presence, as well as extent, of active CNS inflammation.

**Fig. 5.**
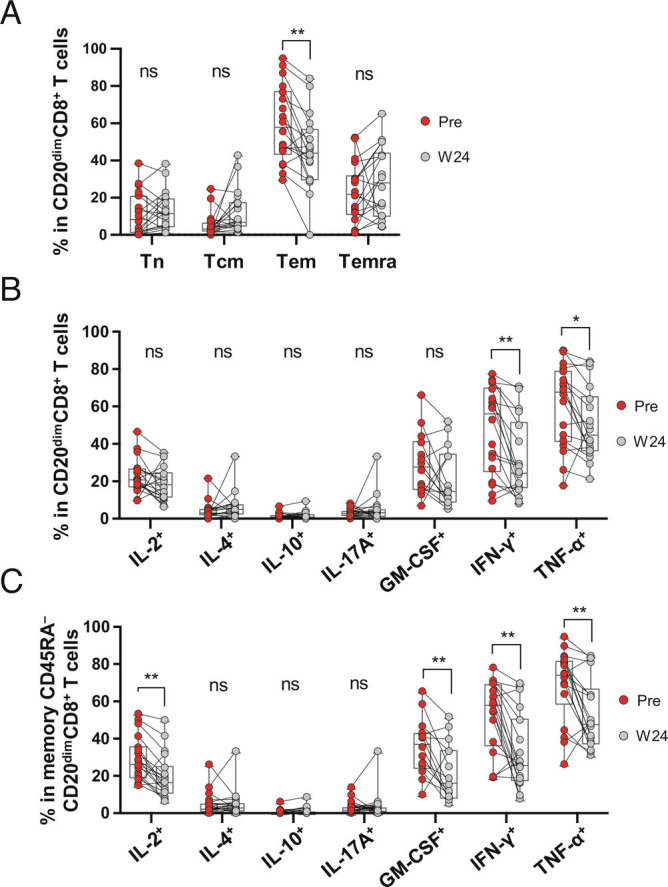
Reappearing CD20^dim^CD8^+^ T cells exhibit a less proinflammatory phenotype compared with pretreatment. Frequencies of naive and memory CD20^dim^CD8^+^ T cells (*A*), cytokine-producing cells CD20^dim^CD8^+^ T cells (*B*), and cytokine-producing memory CD45RA^−^CD20^dim^CD8^+^ T cells (*C*) prior to treatment (Pre) and at time of partial reconstitution at week 24 (W24, n = 18). Statistical analyses were performed by the Wilcoxon matched-pairs signed-rank test followed by multiple comparison correction using the false discovery rate by Benjamini, Krieger, and Yekutieli's two-stage step-up method. *P* value, *P < 0.05, **P < 0.01.

We next considered disease activity emerging following aCD20 treatment initiation. Of a total of 35 patients followed up prospectively with serial assessments, none experienced clinically evident new disease activity, while six patients developed new MRI disease activity defined as the presence of one or more new Gd+ lesions on any given scan at week 12 or thereafter, and/or development of new/enlarging T2 lesions between week 12 scans and subsequent scans (*SI Appendix*, Table S3). We noted that, at baseline, these six patients also had a higher number of Gd+ lesions (*P* < 0.001), in keeping with the known phenomenon wherein pretreatment disease activity is a predictor of on-treatment disease activity, including with ocrelizumab treatment (40, 41). At baseline, these six patients also had a higher number and volume of T2-hyperintense lesions (*P* = 0.011, *P* = 0.050, respectively), and higher numbers of both T cells (*P* = 0.011) and B cells (*P* = 0.047) in their pretreatment CSF (*SI Appendix*, Table S3). Frequencies of circulating CD20-expressing CD8^+^ T cells at baseline (which were strongly inversely associated with the presence of brain MRI Gd+ lesions at baseline) were also associated with new brain lesions developing from week 12 following aCD20 treatment initiation (Fig. 4*G*). There were no appreciable differences in frequencies of CD20-expressing T cells between patients who did or did not develop new disease activity, when measured at either week 12 (when CD20-expressing cells are relatively depleted; Fig. 4*H*) or at week 24 (when CD20-expressing cells can be seen to partially reconstitute; *SI Appendix*, Fig. S6 *A* and *B* and Fig. 4*I*). This “end-of-dose” phenomenon of partial reconstitution of CD20^dim^CD8^+^ T cells in the absence of new disease activity, may reflect the reduced proinflammatory profile of the reconstituting cells, as shown in Fig. 5. In particular, the reemerging CD20^dim^CD8^+^ T cells harbored lower frequencies of effector memory cells (Tem; Fig. 5*A*) and exhibited significantly reduced expression of the proinflammatory cytokines IFN-γ and TNF-α (Fig. 5*B*). This was also evident when assessing the subset of reconstituting memory CD20^dim^CD8^+^ T cells that expressed significantly lower levels of multiple proinflammatory cytokines (IFN-γ, TNF-α, as well as GM-CSF; Fig. 5*C*) compared with pretreatment.

**Fig. 6.**
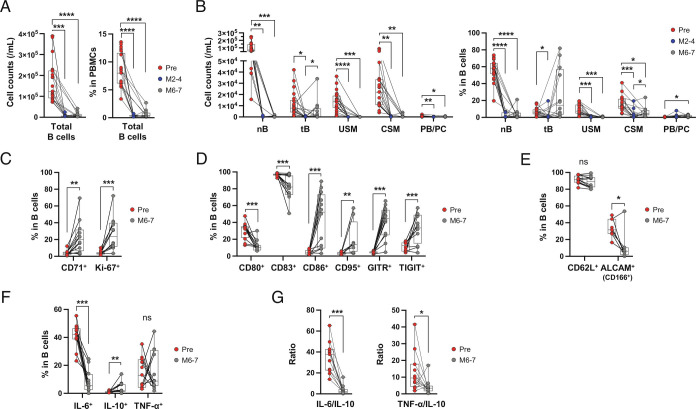
Reappearing B cells early after aCD20 initiation exhibit a predominantly transitional anti-inflammatory phenotype. (*A*) Kinetics of cell counts and frequencies of total B cells at baseline (Pre, n = 21), 2 to 4 mo (M2–4, n = 11) and 6 to 7 mo (M6–7, n = 12) after the first infusion of ocrelizumab. (*B*) Kinetics of cell counts and frequency of B cell subsets in PBMCs. (*C*–*E*) Comparisons of proliferation markers (*C*), costimulatory/coinhibitory molecules (*D*) and adhesion molecules (*E*) expressed on B cells between at Pre and M6–7. (*F*) Comparison of cytokine-producing B cells after ex vivo stimulation with phorbol 12-myristate 13-acetate and ionomycin for 4 h between Pre and M6–7. (*G*) The ratios of proinflammatory cytokines (IL-6 and TNF-α) to anti-inflammatory cytokine (IL-10) in 11 MS patients at Pre and M6–7. Statistical analysis was performed by fitting mixed-effects model with the Geisser–Greenhouse correction, followed by Sidak's multiple comparison to compare the differences among all pairs, or by the Wilcoxon matched-pairs signed-rank test. *P* value, *P < 0.05, **P < 0.01, ***P < 0.001, ****P < 0.0001.

Since reconstitution of proinflammatory B cells represents another mechanism that could theoretically contribute to development of the new disease activity, we observed in the six patients after aCD20 treatment initiation, we investigated the phenotypic and functional profiles of reconstituting B cell subsets (Fig. 6). The near-complete depletion of circulating B cells observed between 2 and 4 mo after aCD20 treatment initiation was followed by limited B cell reconstitution as assessed prior to the next infusion (between 6 and 7 mo following the initial treatment; Fig. 6*A*). Counts of reemerging B cells correlated, as expected, with duration from the prior aCD20 treatment (r = 0.2930; *P* = 0.0256, *SI Appendix*, Fig. S10). Counts and frequencies of class-switched and unswitched memory B cells, plasmablasts and plasma cells remained diminished, with reconstituting B cells largely comprised of CD24^hi^CD38^hi^ transitional B cells and to a much lesser extent mature (CD27^−^IgD^+^) naïve B cells and B cells with a class-switched memory (CD27^+^IgD^−^) phenotype (Fig. 6*B*). Compared with pretreatment B cell profiles, detailed analysis of the reconstituting B cells indicated that they were more proliferative (*P* = 0.001 for CD71, *P* < 0.0001 for Ki-67), expressed lower levels of CD80 (*P* = 0.002), CD83 (*P* < 0.001) and activated leukocyte cell adhesion molecule (ALCAM) (*P* = 0.031), but strikingly higher levels of CD86 (*P* < 0.001), CD95 (*P* = 0.005), the glucocorticoid-induced TNF receptor-related protein (GITR, *P* < 0.001) and TIGIT (*P* = 0.001; Fig. 6 *C*–*E*). Cytokine-expression profiles of the reconstituting B cells were notable for marked decreases in frequencies of IL-6^+^ B cells and increases in frequencies of IL-10^+^ B cells (Fig. 6*F*), overall resulting in decreased ratios of proinflammatory to anti-inflammatory (IL-6/IL-10 and TNF-α/IL-10) cytokine-expressing B cells, compared with their pretreatment profiles (Fig. 6*G*). Overall the reconstituting B cells were characterized by transitional B cell-predominant, more proliferative, less proinflammatory, and more anti-inflammatory cells than pretreatment B cells. In keeping with this profile, we found no association between the reemerging B cell subsets and the limited development of new MS disease activity observed following aCD20 treatment initiation (*SI Appendix*, Fig. S11).

## Discussion

The premise of our study was that the infrequent development of new MS disease activity following initiation of aCD20 may provide a unique window of opportunity to examine how, and potentially when, particular immune-cell subsets contribute to MS relapse biology. The ability of aCD20 treatment to otherwise robustly limit new relapsing MS disease activity, presumably by diminishing disease-implicated T cell responses, could theoretically reflect a direct effect (i.e., eliminating proinflammatory T cells that themselves express CD20) or an indirect effect (i.e., removing B cells that, when present, would contribute to proinflammatory T cell responses). While prior studies have tended to focus on either of these possibilities, they are not mutually exclusive, and in the current study, we had the opportunity to consider both in the same patient cohorts. Through standardized implementation of a comprehensive flow cytometry platform to high-quality samples obtained pretreatment and early post-treatment from independent discovery and validation patient cohorts, we highlight a potential early role for CD20^dim^ T cells (particularly CD20^dim^CD8^+^ T cells) in the development of relapsing MS disease activity. We also describe the differential impact of aCD20 on CD4^+^ and CD8^+^ T cell subsets that provides insights into in vivo interactions between B cells and distinct T cell subsets.

We considered several potential explanations for the infrequent disease activity observed early after aCD20 initiation. Since, in prior studies (5, 6), the same patients experiencing early disease activity did not exhibit further disease activity with continued aCD20 treatment, such early post-treatment disease activity is unlikely to reflect patients whose MS is biologically refractory to aCD20 treatment. It is now known that initial aCD20 doses may not achieve the depth and breadth of cell depletion achieved with subsequent ongoing administration (42–44), hence it is possible that insufficient initial depletion of pathogenic cells in some patients could explain the early disease activity seen after aCD20 treatment initiation. One cannot exclude such a possibility since the assessment of cellular profiles in the circulation is unlikely to be sensitive to varying depths and breadths of tissue depletion. We also considered whether this early disease activity was associated with the reconstitution of proinflammatory B cells and, while there is a similar challenge that tissue reconstitution (potentially relevant to disease activity) may occur prior to the reconstituting cells appearing in the circulation, our data revealed that B cells repopulating the circulation after the initial cycle of aCD20 treatment exhibited more of an anti-inflammatory profile and no apparent relationship to this early disease activity. Specifically, we observed a transitional B cell-predominant phenotype of early repopulating B cells that expressed the activation markers CD86, CD95, and CD71, as well as the Ki67 proliferation marker, with higher levels of IL-10 expression and diminished ratios of proinflammatory to anti-inflammatory (e.g., IL-6/IL-10 and TNF-α/IL-10) cytokines. These repopulating B cells also expressed substantially decreased levels of ALCAM that we previously identified as an important adhesion molecule involved in CNS-trafficking of proinflammatory B cells (38), and higher levels of TIGIT and GITR, which, together with their less proinflammatory cytokine profile, would be expected to have a diminished capacity to induce proinflammatory T cell responses (20, 22, 23, 45, 46). We note that a recent study by Nissimov et al. characterizing B cells repopulating following treatment with the aCD20 rituximab (47), also observed preferential emergence of B cells with an activated naïve/transitional phenotype, though the repopulating B cells in their study exhibited increased IL-6 expression, while we observed decreased frequencies of IL-6-expressing B cells. Thus, while the two studies both identify predominant repopulation by transitional B cells with an activated phenotype, there may be differences in aspects of the functional profile of the reconstituting cells. One potential explanation for the differences may relate to prior immune therapy: all patients in our discovery cohort were DMT-naïve, and among the subset of patients in our validation cohort who were not DMT-naïve, the majority had been on glatiramer acetate or IFN-β, whereas in the Nissimov study 20% of patients were DMT-naïve, and approximately half of all patients were on fingolimod or azathioprine prior to switching to aCD20 treatment. Both fingolimod and azathioprine are known to alter B cell profiles, which would impact the pre-aCD20 baseline samples to which follow-up samples are compared. Another difference between the cohorts is that follow-up samples used in our study were all obtained between 6 and 7 mo after aCD20 initiation, while follow-up samples in the Nissimov study were obtained between 8 and 24 mo after aCD20 initiation. While our focus was on early reconstitution, it is possible that longer duration of treatment influences the depth and breadth of depletion and that longer periods of reconstitution are associated with evolving functional profiles of the reemerging B cells. One also notes that the Nissimov study evaluated patients treated with rituximab, while we studied patients treated with ocrelizumab and it is interesting to speculate on whether the somewhat different repopulation profiles may reflect differences in the depth and breadth of B cell subset depletion (and subsequent reconstitution) with ocrelizumab versus rituximab. While we did not document a relationship between reconstituting B cell subsets and the disease activity observed early after aCD20 initiation, this does not preclude such a mechanism, and it is possible that if B cells are allowed to reconstitute further and over longer periods, some patients will experience new relapsing activity on that basis, possibly when repopulation includes proinflammatory memory B cells rather than predominantly naive/transitional B cells, as described in the context of neuromyelitis optica and rheumatoid arthritis (48–51).

Another possible explanation for the disease activity observed early after aCD20 initiation in our study is that treatment was instituted in these few individuals with relapse biology already in progress (and downstream of where aCD20 exercises its therapeutic mode of action). Our findings of an inverse relationship between pretreatment levels of circulating CD20^dim^ T cells (especially CD8^+^ T cells) and CNS inflammatory disease activity observed both prior to and following initiation of aCD20 treatment is most in keeping with the latter scenario and the possibility that CD20-expressing T cells transition out of the circulation to participate in the early development of new disease activity in one or more disease-relevant compartment/s. This would be analogous to the prior observation that treatment with natalizumab (anti-VLA4, that acts principally by preventing trafficking of cells to the disease target organ) was not beneficial when administered after relapse onset (52). In contrast, natalizumab is highly effective at preventing the development of new relapses and was noted to increase the frequency of circulating CD20-expressing T cells (30, 53), likely a reflection that these cells were no longer able to egress from the circulation into the tissues, and highlighting how different MS therapies can differentially impact the same disease-implicated immune cells (27, 30, 31, 53). We confirm and extend prior work characterizing CD20-expressing T cells in MS patients (28–31, 53, 54) with the demonstration that they harbor a highly proinflammatory and CNS-migratory profile. Our speculation that these cells transition from the blood into the CNS as part of active CNS inflammation is supported by several previous observations, including pathology studies reporting that active MS brain lesions contain increased numbers of CD8^+^ T cells characterized as tissue-resident memory CD8^+^ T cells or mucosal-associated invariant T (MAIT) cells (54–59), and enriched for CD20-expressing T cells (54, 60). Although our cohort did not include serial CSF analysis, increased numbers of CD3^+^CD20^dim^ cells have also been identified in the CSF of MS patients where they are reportedly positively correlated with disease severity (27). Of interest for future studies will be to directly investigate CD20-expressing T cells in the circulation and CSF of patients, and correlate their levels with disease activity. We speculate that if the presence of these cells in the CNS over time is predicated on their trafficking from the periphery, then one would expect them to be diminished there with ongoing aCD20 therapy. This would reflect at least in part the direct removal of the CD20-expressing T cells from the circulation but possibly also a decrease in the “de novo” generation of CD20-expressing T cells whose surface-expression of CD20 may rely on CD8 T cells interacting with B cells, for example via trogocytosis (31). Overall, our findings implicate CD20-expressing T cells, especially CD8^+^ T cells, in early MS relapse biology and imply that the impact of aCD20 initiation could depend on the timing with which treatment is instituted relative to already developing relapse biology.

Our findings also raise the question as to whether levels of CD20-expressing CD8^+^ T cells in patients with MS may be predictive of CNS inflammatory disease activity. However, in addition to further validation in larger cohorts, more biomarker development work will be required (for example, to better define the ranges of CD20-expressing CD8^+^ T cell frequencies associated with different disease-activity outcomes or whether a particular cutoff could be identified) to establish whether and how measuring frequencies of these cells may be clinically useful. There may also be important differences in the utility of measuring frequencies of CD20-expressing T cells prior to initiation of aCD20 therapy, versus measuring them during aCD20 therapy. While our findings suggest that pretreatment frequencies of these cells may prove to be predictive of disease activity, we also show that once on aCD20 treatment, CD20-expressing T cells reappear in the circulation during early reconstitution yet are not associated with new disease activity, which may be explained by our demonstration that they reemerge with a lesser proinflammatory or CNS-homing phenotype. This may mean that to be useful, any meaningful assessment of CD20-expressing T cells will require not only measuring their frequency but also some aspects of their functional phenotype.

Treatment with aCD20 offers an added opportunity to examine how B cells interact in vivo with T cells in humans, and particularly whether these interactions differ for distinct T cell subsets—of interest not just in the context of MS and MS therapeutics but also in the autoimmune disease field more generally as well as in the general population. For example, a recent severe acute respiratory syndrome coronavirus 2 (SARS-CoV-2) vaccine study revealed that vaccine-specific responses of CD4^+^ T cells were mildly attenuated, while vaccine-specific responses of CD8^+^ T cells were enhanced in patients with MS treated with aCD20 (61). While the diminished CD4^+^ T cell responses could theoretically be explained by the removal of CD20-expressing CD4^+^ T cells, the increased CD8^+^ T cell responses in aCD20-treated patients could not be explained on the basis of removing CD20-expressing CD8^+^ T cells, and must reflect some previously unappreciated in vivo interaction between CD8^+^ T cells and B cells (or the antibodies they can be driven to produce). Our study also enables us to comment on the differential in vivo impact of aCD20 treatment on CD4^+^ and CD8^+^ T cell subsets. We confirm and extend prior reports noting that aCD20 therapy reduces both CD4^+^ and CD8^+^ effector memory, proinflammatory cytokine-producing and CNS-trafficking T cells, though our focus on CD20-expressing T cells suggests that the mechanisms by which aCD20 treatment impacts CD4^+^ and CD8^+^ T cells are different. We note that direct removal of pretreatment proinflammatory CD20-expressing CD8^+^ T cells had a greater contribution to treatment-associated changes in the CD8^+^ T cell pool than was the case for CD4^+^ T cells. This adds to prior reports suggesting that the impact of aCD20 on proinflammatory CD4^+^ T cell responses reflects (at least in part), the indirect consequence of depleting proinflammatory B cells and thus disrupting in vivo B cell-CD4^+^ T cell interactions that would otherwise drive proinflammatory T cell responses as suggested in prior work (16, 21, 22). The observation that B cell-targeting with anti-CD19 (aCD19) also limits MS relapsing disease activity (62) makes it unlikely that the removal of CD20-expressing T cells provides the full explanation for the therapeutic mode of action of aCD20 in patients with relapsing MS, although it is possible that aCD19 therapy could indirectly reduce CD20^dim^ T cells for example by preventing B cell-T cell contact with potential trogocytosis of CD20 molecules from B cells to the T cells (31, 63).

There are several limitations to our study and questions that remain to be elucidated. As new disease activity develops only infrequently after aCD20 initiation, none of our patients experienced a documented clinical relapse. Similarly, the Expanded Disability Status Scale (EDSS) remained essentially stable, with only three patients in the validation cohort having a marginal change in documented EDSS scores. Thus, our study was underpowered to assess the associations between particular immune-cell subsets and clinically evident disease activity. We were interested to note that compared with CD20-expressing B cells that were almost all depleted in the circulation early after aCD20 treatment, depletion of CD20^dim^ T cells appeared less complete, which may reflect their lower levels of CD20 expression that may make them less susceptible to depletion through complement-dependent cytotoxicity and/or antibody-dependent cellular cytotoxicity). The CD20^dim^ T cells also appeared to reconstitute faster than the B cells, which could reflect a lesser depth of depletion or potentially ongoing encounters of T cells with incompletely depleted B cells deeper in tissues, and acquisition of CD20 by trogocytosis (31). Future studies with larger numbers will also help assess whether the reemergence of CD20-expressing T cells would be associated with new disease activity, though it is possible that the lack of such an association reflects a lack of relevant B cell-T cell interactions (i.e., potentially pathogenic T cells may reconstitute but need to be triggered by proinflammatory B cells).

In summary, our phenotypic and functional characterization of circulating immune-cell subsets in independent discovery and validation cohorts in the context of aCD20 treatment initiation in MS patients and our examination of the association between immune-cell subsets and measures of disease activity observed both prior to and early following aCD20 treatment initiation provides insights into distinct in vivo interactions between B cells and CD4^+^ versus CD8^+^ T cell subsets, and implicates CD20-expressing T cells (and particularly CD8^+^ T cells) in developing MS disease activity. We emphasize that disease activity in patients treated with aCD20 may emerge for very different biological reasons. In this study of the early disease activity infrequently seen following aCD20 treatment initiation, our findings indicate that this disease activity in fact is not related to a failure of the aCD20 therapy itself, but rather reflects the presence of ongoing relapse biology that is not immediately targeted by aCD20, and is consistent with a particularly early role of CD20-expressing T cells in newly developing relapsing MS disease activity. The emergence of disease activity in patients after aCD20 treatment-initiation could include insufficient depth and breadth of initial depletion of CD20-expressing cells such that several cycles or higher doses of aCD20 treatment may be required to fully abrogate new relapse biology. It is likely that “true aCD20 nonresponders” (defined as those manifesting new relapsing disease activity when fully B cell depleted) are relatively rare. Another context in which disease activity may emerge while on aCD20 treatment could be reconstitution of B cells as seen in other autoimmune conditions treated with aCD20 therapy such as neuromyelitis optica spectrum disorders (48, 49), rheumatoid arthritis (50, 51), lupus (64), and pemphigus (65, 66), apparently driven by a return of pathogenic proinflammatory memory B cells. Similar to reappearing CD20^dim^CD8^+^ T cells, the early repopulating B cells that we document in our study using ocrelizumab appear less proinflammatory than B cells recently noted to repopulate early after treatment with rituximab. While longer reconstitution following ocrelizumab may be associated with reemergence of proinflammatory memory B cells in some MS patients, it is interesting to speculate whether our findings may reflect different depths and/or breadths of B cell depletion and subsequent reconstitution with different aCD20 treatments. Of particular interest for future studies is whether durable disease quiescence may be achieved following transient aCD20 treatment and long-term B cell reconstitution in a subset of patients and whether this will depend on the initial depth and breadth of depletion of CD20-expressing B and T cells and their profiles of reconstitution.

## Materials and Methods

For deep immune phenotyping using multiparametric flow cytometry, we studied MS patients treated with ocrelizumab in two independent cohorts (Table 1). In both cohorts, all MS patients were diagnosed by the 2010 McDonald criteria (67). In the first "discovery" cohort, 23 DMT-naive patients with MS were prospectively enrolled at a single MS center, the Penn Comprehensive MS center, Hospital of the University of Pennsylvania (*SI Appendix*, Table S1). In the second "validation" cohort, 35 patients were enrolled as part of an open-label, multicenter, biomarker study of MS patients initiating ocrelizumab. All patients were diagnosed as having RRMS and subject to neurological evaluation, blood sampling, and MRI imaging at baseline, weeks 12, 24, and 52 after the first infusion of ocrelizumab (*SI Appendix*, Table S2). None of the patients received systemic steroids within at least 4 wk of any blood sampling. Written informed consent was obtained from all donors. This study was approved by the Institutional Review Board of the University of Pennsylvania and conducted according to the World Medical Association Declaration of Helsinki. Detailed descriptions of study materials and methods are provided in *SI Appendix*, *SI Materials and Methods*.

## Supplementary Material

Appendix 01 (PDF)

## Data Availability

All study data are included in the article and/or *SI Appendix*.
