# Supplementary material for: Differential effects of anti-CD20 therapy on CD4 and CD8 T cells and implication of CD20-expressing CD8 T cells in MS disease activity
Source: Proc Natl Acad Sci U S A. 2023 Jan 12;120(3):e2207291120. doi: 10.1073/pnas.2207291120 (PMC9934304; doi:10.1073/pnas.2207291120)
Supplement: Supplementary file 1 — Appendix 01 (PDF) [file pnas.2207291120.sapp.pdf]

PNAS
www.pnas.org

Supplementary Information for

Differential effects of anti-CD20 therapy on CD4 and CD8 T cells and implication of CD20-expressing CD8 T cells in MS disease activity

Koji Shinoda[1,2], Rui Li[1,2], Ayman Rezk[1,2], Ina Mexhitaj[1,2], Kristina R. Patterson[1,2], Mihir Kakara[1,2], Leah Zuroff[1,2], Jeffrey L. Bennett[3], H.-Christian von Büdingen[4], Robert Carruthers[5], Keith R. Edwards[6], Robert Fallis[7], Paul S. Giacomini[8], Benjamin M. Greenberg[9], David A. Hafler[10], Carolina Ionete[11], Ulrike W. Kaunzner[12], Christopher B. Lock[13], Erin E. Longbrake[14], Gabriel Pardo[15], Fredrik Piehl[16,17,18], Martin S. Weber[19,20], Tjalf Ziemssen[21], Dina Jacobs[1,2], Jeffrey M. Gelfand[22], Anne H. Cross[23], Briana Cameron[24], Bruno Musch[24], Ryan C. Winger[24], Xiaoming Jia[24], Christopher T. Harp[24], Ann Herman[24], Amit Bar-Or[1,2,25*]

[1] Department of Neurology, Perelman School of Medicine, University of Pennsylvania, Philadelphia, PA, USA.
[2] Center for Neuroinflammation and Experimental Therapeutics, Perelman School of Medicine, University of Pennsylvania, Philadelphia, PA, USA.
[3] Departments of Neurology and Ophthalmology, Programs in Neuroscience and Immunology, University of Colorado School of Medicine, Aurora, CO, USA.
[4] F. Hoffmann-La Roche, Basel, Switzerland.
[5] Department of Medicine, University of British Columbia, Vancouver, BC, Canada.
[6] MS Center of Northeastern New York, Comprehensive MS Care Center Affiliated with the National MS Society, Latham, NY, USA.
[7] Department of Neurology, Ohio State University Medical Center, Columbus, OH, USA.
[8] Department of Neurology and Neurosurgery, Montreal Neurological Institute, McGill University, Montreal, QC, Canada.
[9] Department of Neurology, University of Texas Southwestern Medical Center, Dallas, TX, USA.
[10] Departments of Neurology and Immunobiology, Yale School of Medicine, New Haven, CT, USA.
[11] Department of Neurology, University of Massachusetts Medical School, Worcester, MA, USA.
[12] Judith Jaffe Multiple Sclerosis Center, Weill Cornell Medicine, New York, NY, USA.
[13] Department of Neurology and Neurological Sciences, Stanford University, Palo Alto, CA, USA.
[14] Department of Neurology, Yale University, New Haven, CT, USA.
[15] Oklahoma Medical Research Foundation, Multiple Sclerosis Center of Excellence, Oklahoma City, OK, USA.
[16] Department of Clinical Neuroscience, Karolinska Institute, Stockholm, Sweden.
[17] Department of Neurology, Karolinska University Hospital, Stockholm, Sweden.
[18] Neuroimmunology Unit, Center for Molecular Medicine, Karolinska University Hospital, Karolinska Institute, Stockholm, Sweden.
[19] Institute of Neuropathology, University Medical Center, Göttingen, Germany.
[20] Department of Neurology, University Medical Center, Göttingen, Germany.

[21] Department of Neurology, Center of Clinical Neuroscience, University Hospital Carl Gustav Carus, Technical University of Dresden, Dresden, Germany.
[22] Weill Institute for Neurosciences, Multiple Sclerosis Center, University of California, San Francisco, CA, USA.
[23] Department of Neurology, Washington University School of Medicine, Saint Louis, MO, USA.
[24] Genentech, Inc., South San Francisco, CA, USA.
[25] Children's Hospital of Philadelphia, University of Pennsylvania, Philadelphia, PA, USA.

***Correspondence to Prof. Amit Bar-Or**, Center for Neuroinflammation and Experimental Therapeutics, Department of Neurology, Multiple Sclerosis Division, Perelman School of Medicine, University of Pennsylvania. Address: 3400 Spruce Street – 3 Gates Building, Philadelphia, PA 19104, USA. Phone: 215-662-3606. E-mail: amitbar@pennmedicine.upenn.edu

This PDF file includes:

Supplementary text 2568 words

Figures S1 to S11

Tables S1 to S6

SI References 2

Supplementary Information Text

SI Materials and Methods

Blood sample processing

In the Discovery cohort, PBMCs were processed using standard operating procedures for all steps of blood procurement, PBMC isolation (by density gradient centrifugation using Ficoll-Paque Plus; GE Healthcare, Little Chalfont, UK), cryopreservation and storage in liquid nitrogen vapor phase, as previously described (1). In the validation cohort, whole blood cell counts were measured using 6-color TBNK Reagent with Trucount tubes (BD, San Diego, CA) and PBMC were separated by density gradient centrifugation using LymphoPrep tubes (Axis Shield, Dunde, UK), frozen and stored in CTL-Cryo ABC Kit Freeze Medium (Cellular Technologies Limited, Shaker Heights, OH) in liquid nitrogen vapor phase by a certified central laboratory (Covance, Indianapolis, IN), with PBMC subsequently sent on dry ice for batched analyses at Penn using strict standardized operating procedures.

Flow cytometric and fluid marker analyses

Thawed PBMC were suspended in serum-free X-VIVO 10 media (Lonza, Basel, Switzerland) and subject to surface staining and intracellular cytokine staining which were standardized for cryopreserved PBMC to characterize the phenotype and functional profiles of immune subsets using antibodies and reagents as shown in Fig. S1 and S2, and Tables S4-6. For surface staining, cells were rested at 37ºC for 4 hours, labeled with Live/Dead Fixable Aqua Dead Cell Stain Kit (Thermo Fisher Scientific, Waltham, MA), stained with antibodies at the optimal concentrations together with Brilliant Stain Buffer (BD Biosciences, Franklin Lakes, NJ) at room temperature for 30 minutes, and fixed using BD Cytofix/Cytoperm Fixation/Permeabilization Solution Kit (BD Biosciences). Chemokine receptors were stained at 37ºC for 15 minutes prior to surface staining of other markers. For intracellular cytokine staining, cells were cultured with 20 ng/ml of phorbol 12-myristate 13-acetate (Sigma-Aldrich, St. Louis, MO) and 500 ng/ml of ionomycin (Sigma-Aldrich) in the presence of GoldiStop (BD Biosciences) for 4 hours at 37°C. Intracellular staining was performed using BD Cytofix/Cytoperm Fixation/Permeabilization Solution Kit or eBioscience Foxp3/Transcription Factor Staining Buffer Set (Thermo Fisher Scientific). Stained cells were analyzed on a FACS Fortessa flow cytometer (BD Biosciences) on the same day. Rainbow Fluorescent Particles (Biolegend, San Diego, CA) were used to standardize the instrument settings and to minimize batch effects across experiments. Serial samples of the same individuals were always run in the same batched experiment. Data were analyzed using FlowJo software (BD Biosciences). As part of quality control, samples not meeting predefined criteria (at least 75% viability measured by Live/Dead staining) were excluded from analysis. Average PBMC sample viabilities were 93.6 % (SD 4.9, range 78.5–98.0) in the Discovery cohort and 92.4% (SD 4.5, range 77.6–98.7) in the Validation cohort. Pre-treatment (baseline) CSF samples were processed within 1 hour of lumbar puncture. All samples were centrifuged at 400xg for 10 minutes at room temperature with supernatant removed and stored at -80°C, until batched analysis for levels of neurofilament light chain (NfL) using Quanterix Simoa NF-light Advantage Kit (Billerica, MA, USA), and neurofilament heavy chain (NfH) as well as glial fibrillary acidic protein (GFAP), using ProteinSimple (San Jose, CA, USA). Fresh CSF cell pellets were resuspended in BD buffer (BD Biosciences, San Jose, CA, USA) and blocked with Fc block (BD Biosciences) for 10 minutes at room temperature. Zombie Aqua fixable viability dye (Biolegend, San Diego, CA, USA) and antibody staining cocktail containing CD3-PerCP-Cy5.5 and CD19-BV421 (Biolegend) were added to the cells and incubated at room temperature for 15 minutes in the dark, with cells then washed with BD buffer, resuspended in 0.5% paraformaldehyde (Electron Microscopy Sciences, Hatfield, PA, USA) and acquired via flow cytometry within 6 hours of collection.

MR imaging and identification of new disease activity

Validation cohort patients underwent standardized brain MRIs at baseline and at weeks 12, 24, and 52 following initiation of ocrelizumab. MRI acquisition protocol included pre- and post-gadolinium (Gd) injection (0.1 mmol/kg, 10-min post-injection delay) axial 3 mm T1-weighted slices (3D spoiled gradient-echo, repetition time = 28–30 ms, echo time = 5–11 ms, flip angle = 27–30), as well as axial 3 mm T2-weighted slices (2D fast spin-echo, repetition time = 4000–6190 ms, echo time = 74–91 ms and echo train length = 7–11), as previously described (2). Centralized reading (NeuroRx) was performed to ascertain the development of new disease activity defined as either presence of new Gd-enhancing T1 lesion/s or appearance of one or more new and/or enlarging T2 lesion/s compared to prior MRI.

Statistical analysis

Statistical analysis was performed using Prism 9 ver. 9.0.0 (GraphPad Software, San Diego, CA) or JMP Pro 15 ver. 15.2.0 (SAS Institute, Cary, NC). Normality was evaluated by Shapiro-Wilk test. Unpaired data were compared by parametric *t*-test and non-parametric Mann-Whitney test. Paired samples were compared using non-parametric Wilcoxon matched-pairs ranked-sum test to compare paired samples followed by multiple comparison correction using False Discovery Rate by Benjamini, Krieger and Yekutieli's two-stage step-up method. The repeated measures data containing missing values were analyzed using a repeated measures mixed-effect model, with the Geisser-Greenhouse correction for sphericity, followed by Holm-Sidak's multiple comparison test. Correlations were examined using Pearson or Spearman correlation coefficient test based on the normality examined by the Shapiro-Wilk test. A p-value < 0.05 was considered statistically significant.

References

1. I. Mexhitaj, *et al.*, Abnormal effector and regulatory T cell subsets in paediatric-onset multiple sclerosis. *Brain* **142**, 617–632 (2019).
2. C. Elliott, *et al.*, Chronic white matter lesion activity predicts clinical progression in primary progressive multiple sclerosis. *Brain* **142**, 2787–2799 (2019).

Figure S1. Representative gating strategy of immune phenotyping

(A) Gating strategy for major immune-cell types. (B) Naive and memory $CD4^+$ and $CD8^+$ T-cell subsets classified using CCR7 and CD45RA. (C) Exhausted $CD8^+$ T cells expressing inhibitory receptors and Eomes. Expression of Eomes in non-naive $CD8^+$ T cells was evaluated in comparison with their background staining in naive $CD8^+$ T cells. (D) CCR2 and CCR5 expression among $CD4^+$ and $CD8^+$ T cells. (E) Adhesion molecule expression by $CD4^+$ and $CD8^+$ T cells. (F) Cytokine-producing $CD4^+$ and $CD8^+$ T cells after *ex vivo* stimulation with phorbol 12-myristate 13-acetate and ionomycin in the presence of GoldiStop for 4 hours. (G) Regulatory $CD4^+$ T cells (Treg) defined as $CD25^{hi}$,$CD127^{lo/–}$, and functional marker expression by Treg. (H) Gating strategy of B-cell subsets. Abbreviations: ALCAM, activated leukocyte cell adhesion molecule; CSM, class-switched memory B cell; GITR, glucocorticoid-induced TNF receptor-related protein; gMFI, geometric mean fluorescent intensity; MCAM, melanoma cell adhesion molecule; nB, naive B cell; PBMC, peripheral blood mononuclear cells; PB/PC, plasmablast/plasma cell; tB, transitional B cell; Tcm, central memory T cell; Tem, effector memory T cell; Temra, terminally differentiated effector memory T cell; TIGIT, T cell immunoglobulin with ITIM domain; Tn, naive T cell; USM, unswitched memory B cell.

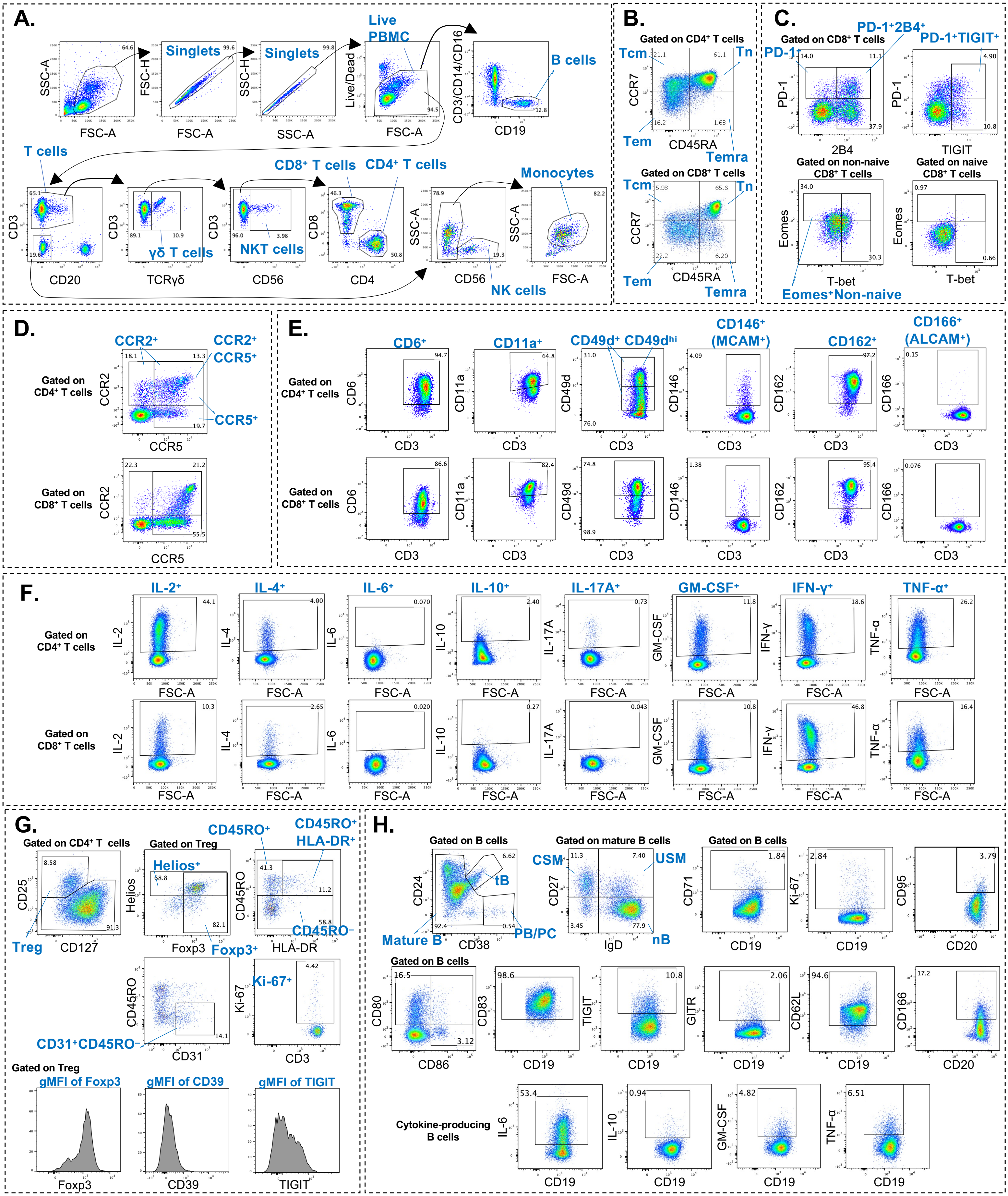

A.
Live PBMC
Singlets
Singlets
B cells
T cells
CD8⁺ T cells
CD4⁺ T cells
Monocytes
γδ T cells
NKT cells
NK cells
SSC-A
FSC-A
FSC-H
SSC-H
Live/Dead
CD3/CD14/CD16
CD19
CD3
CD20
TCRγδ
CD56
CD8
CD4
B.
Gated on CD4⁺ T cells
Gated on CD8⁺ T cells
Tcm
Tn
Tem
Temra
CCR7
CD45RA
C.
Gated on CD8⁺ T cells
PD-1⁺
PD-1⁺2B4⁺
PD-1⁺TIGIT⁺
PD-1
2B4
TIGIT
Gated on non-naive CD8⁺ T cells
Gated on naive CD8⁺ T cells
Eomes
T-bet
Eomes⁺Non-naive
D.
Gated on CD4⁺ T cells
Gated on CD8⁺ T cells
CCR2⁺
CCR2⁺ CCR5⁺
CCR5⁺
CCR2
CCR5
E.
Gated on CD4⁺ T cells
Gated on CD8⁺ T cells
CD6⁺
CD11a⁺
CD49d⁺ CD49dʰⁱ
CD146⁺ (MCAM⁺)
CD162⁺
CD166⁺ (ALCAM⁺)
CD6
CD11a
CD49d
CD146
CD162
CD166
CD3
F.
Gated on CD4⁺ T cells
Gated on CD8⁺ T cells
IL-2⁺
IL-4⁺
IL-6⁺
IL-10⁺
IL-17A⁺
GM-CSF⁺
IFN-γ⁺
TNF-α⁺
IL-2
IL-4
IL-6
IL-10
IL-17A
GM-CSF
IFN-γ
TNF-α
FSC-A
G.
Gated on CD4⁺ T cells
Gated on Treg
CD45RO⁺
CD45RO⁺ HLA-DR⁺
Helios⁺
Treg
Foxp3⁺
CD45RO⁻
Ki-67⁺
CD31⁺CD45RO⁻
CD25
CD127
Helios
Foxp3
CD45RO
HLA-DR
CD31
Ki-67
CD3
gMFI of Foxp3
gMFI of CD39
gMFI of TIGIT
CD39
TIGIT
H.
Gated on B cells
Gated on mature B cells
CSM
USM
tB
Mature B
PB/PC
nB
CD24
CD38
CD27
IgD
CD71
CD19
Ki-67
CD95
CD20
CD80
CD86
CD83
TIGIT
GITR
CD62L
CD166
Cytokine-producing B cells
IL-6
IL-10
GM-CSF
TNF-α

Figure S2. Anti-CD20 treatment reduces CD8$^+$ T-cell subsets associated with exhaustion

Changes in absolute counts and frequencies of exhausted-cell markers expressed by CD8$^+$ T cells between pre-treatment (Pre) and 2-4 months (M2-4) after anti-CD20 (ocrelizumab) treatment initiation (n = 10). Statistical analysis was performed by Wilcoxon matched-pairs signed-rank test. *p* value, **p* < 0.05, ***p* < 0.01, ****p* < 0.001.

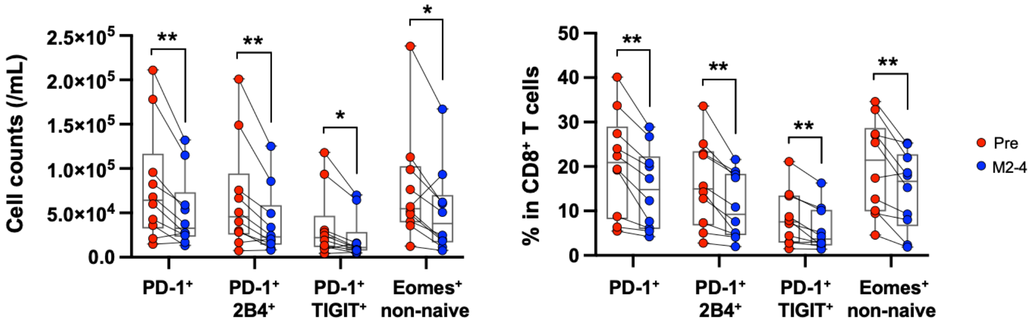

Figure S3. No impact of anti-CD20 treatment initiation on regulatory CD4⁺ T-cell subsets

(A) Changes in absolute counts and frequencies of phenotypically-defined regulatory CD4⁺ T cells (Treg, CD3⁺CD4⁺CD25⁺CD127lo/–) cells in MS patients between pre-treatment (Pre, n = 13) and 2-4 months after anti-CD20 (ocrelizumab) treatment initiation (M2-4, n = 10). (B) Changes in frequencies of Treg subsets including naive (CD45RO⁻), memory (CD45RO⁺), Helios⁺, CD39⁺, activated (HLA-DR⁺CD45RO⁺), proliferating (Ki-67⁺) and recent thymic emigrant (RTE; CD31⁺CD45RO⁻) Tregs. (C) Changes in geometric mean fluorescent intensity (MFI) of CD4⁺ Treg markers, Foxp3, CD39 and TIGIT. Statistical analysis was performed by Wilcoxon matched-pairs signed-rank test. NS, not significant.

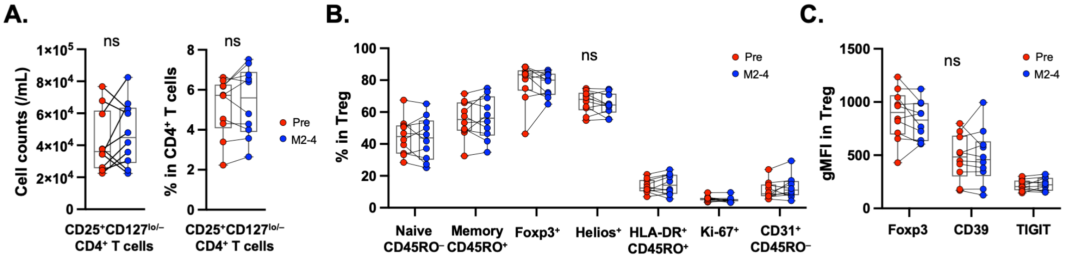

Figure S4. Replication of immune-cell subset changes following anti-CD20 treatment - Validation cohort

Thirty-five patients with relapsing-remitting multiple sclerosis (RRMS) were enrolled in the validation cohort. Blood sampling was serially performed at baseline (W0), week 12 (W12) and week 24 (W24) after initiation of ocrelizumab. Whole blood cell counts were analyzed in all patients and cryopreserved peripheral blood mononuclear cells subsequently underwent comprehensive functional phenotyping using multi-parametric flow cytometry. (A) Major lymphocyte population counts in whole blood at W0, W12 and W24. (B) CD4/CD8 ratio in whole blood. (C) Absolute cell counts and (D) frequencies of naive and memory cells among $CD4^+$ and $CD8^+$ T cells, respectively. (E) Cell counts and (F) frequencies of cytokine-producing $CD4^+$ and $CD8^+$ T cells, respectively, after *ex vivo* stimulation with phorbol 12-myristate 13-acetate and ionomycin for 4 hours. (G) Counts and frequencies of $CCR2^+$ $CCR5^+CD4^+$ and (H) $CCR2^+$ $CCR5^+$ $CD8^+$ T cells. (I) Counts and frequencies of adhesion molecule-expressing $CD4^+$ and (J) $CD8^+$ T cells . Statistical analysis was performed by fitting the mixed-effects model with the Geisser-Greenhouse correction (accounting for missing data and correcting the violation of sphericity), followed by Sidak's multiple comparison to compare the differences among all pairs. *p* value, *$p < 0.05$, **$p < 0.01$, ***$p < 0.001$, ****$p < 0.0001$.

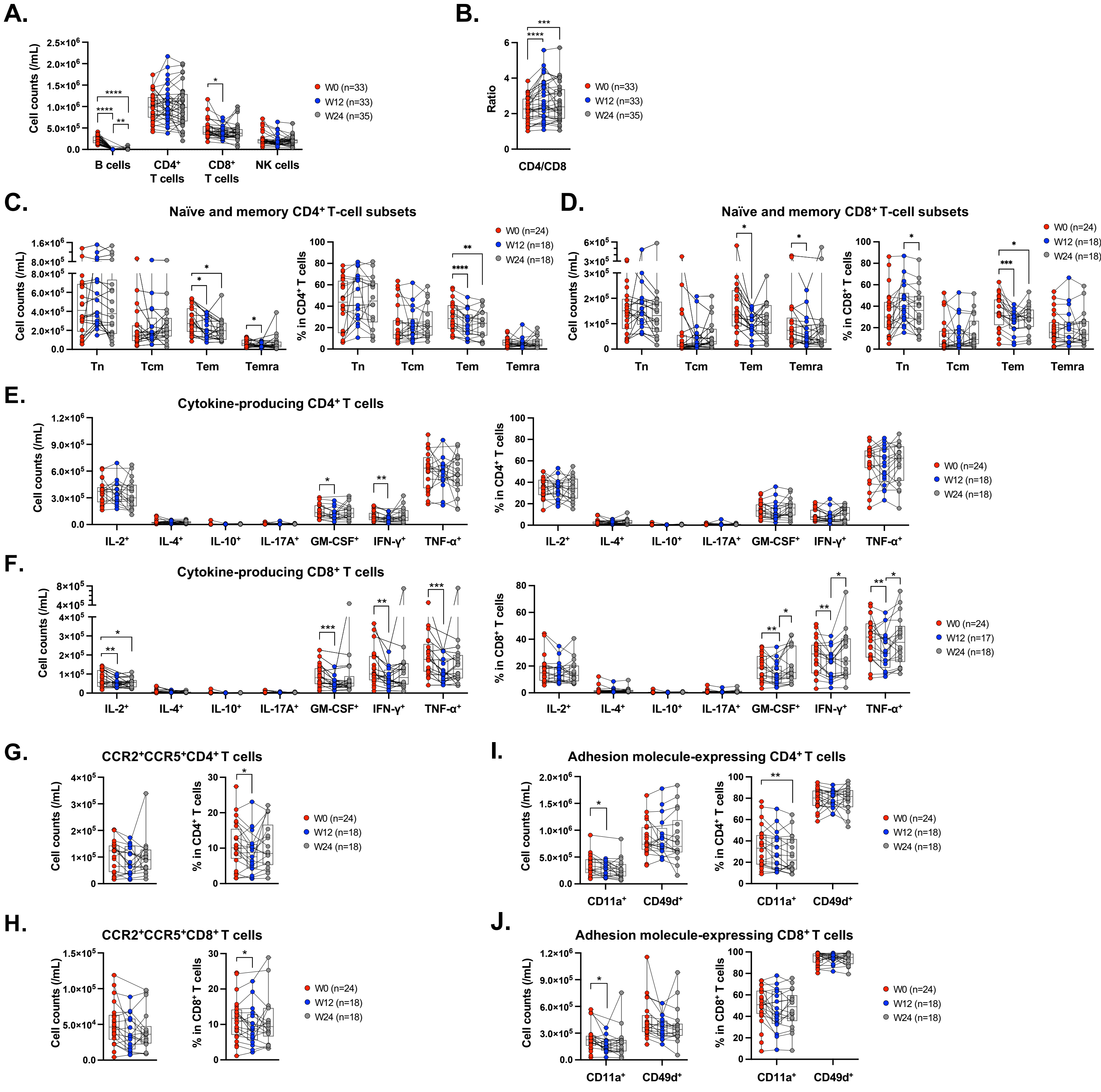
A.
Cell counts (/mL)
B cells
CD4+ T cells
CD8+ T cells
NK cells
W0 (n=33)
W12 (n=33)
W24 (n=35)
B.
Ratio
CD4/CD8
W0 (n=33)
W12 (n=33)
W24 (n=35)
C.
Naïve and memory CD4+ T-cell subsets
Cell counts (/mL)
% in CD4+ T cells
Tn
Tcm
Tem
Temra
W0 (n=24)
W12 (n=18)
W24 (n=18)
D.
Naïve and memory CD8+ T-cell subsets
Cell counts (/mL)
% in CD8+ T cells
Tn
Tcm
Tem
Temra
W0 (n=24)
W12 (n=18)
W24 (n=18)
E.
Cytokine-producing CD4+ T cells
Cell counts (/mL)
% in CD4+ T cells
IL-2+
IL-4+
IL-10+
IL-17A+
GM-CSF+
IFN-γ+
TNF-α+
W0 (n=24)
W12 (n=18)
W24 (n=18)
F.
Cytokine-producing CD8+ T cells
Cell counts (/mL)
% in CD8+ T cells
IL-2+
IL-4+
IL-10+
IL-17A+
GM-CSF+
IFN-γ+
TNF-α+
W0 (n=24)
W12 (n=17)
W24 (n=18)
G.
CCR2+CCR5+CD4+ T cells
Cell counts (/mL)
% in CD4+ T cells
W0 (n=24)
W12 (n=18)
W24 (n=18)
H.
CCR2+CCR5+CD8+ T cells
Cell counts (/mL)
% in CD8+ T cells
W0 (n=24)
W12 (n=18)
W24 (n=18)
I.
Adhesion molecule-expressing CD4+ T cells
Cell counts (/mL)
% in CD4+ T cells
CD11a+
CD49d+
W0 (n=24)
W12 (n=18)
W24 (n=18)
J.
Adhesion molecule-expressing CD8+ T cells
Cell counts (/mL)
% in CD8+ T cells
CD11a+
CD49d+
W0 (n=24)
W12 (n=18)
W24 (n=18)

Figure S5. Identification of CD20^{dim} T cells and their depletion by ocrelizumab

(A) Representative example of staining for CD20^{dim} T cells, gated on live PBMCs. CD20^{dim} T cells were gated in comparison with negative control of fluorochrome minus one (FMO). (B) Representative images of CD20^{dim} T cells at baseline (Pre-ocrelizumab) and 2-4 months (Post-ocrelizumab) after the first infusion of ocrelizumab. (C) Representative images of CD3^{+} T cells expressing surface CD20 (clone 2H7) and cytoplasmic CD20 (clone 1412) in an MS patient, before and after ocrelizumab treatment. (D) Changes in frequencies of total CD19 expressing B cells in MS patient (n = 4) PBMC pre- and post-ocrelizumab treatment demonstrating effective depletion. (E) Changes in frequencies of surface and cytoplasmic CD20-expressing T cells, before and after ocrelizumab treatment. Abbreviations: FMO, fluorochrome minus one.

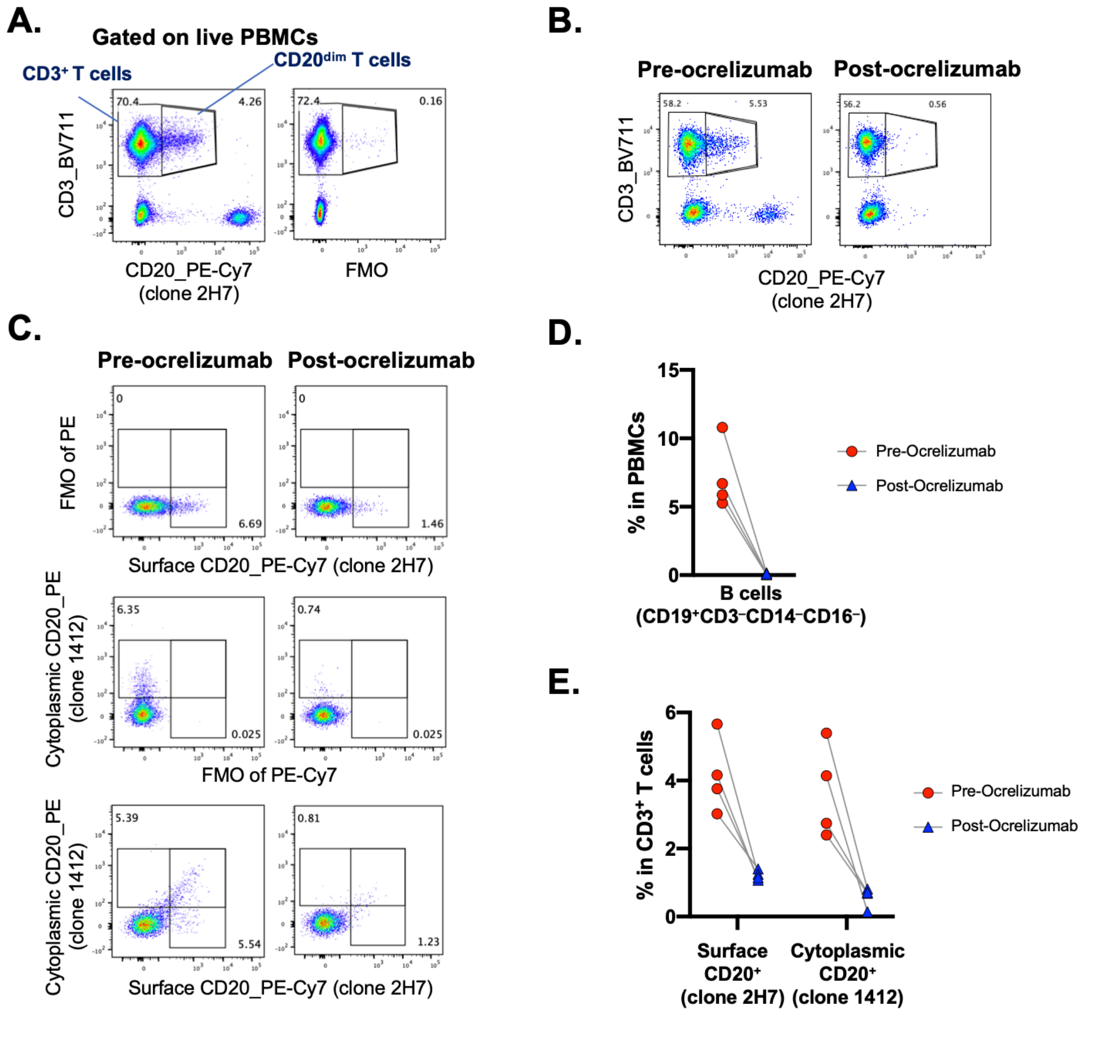

Figure S6. Replication of anti-CD20-induced depletion of $CD20^{dim}$ T cells - Validation cohort

(A) Cell counts and frequencies of $CD20^{dim}$ T cells Pre-treatment (W0), and week 12 (W12) and week 24 (W24) after the first infusion of ocrelizumab. (B) Cell counts and frequencies of $CD20^{dim}$ cells among $CD4^+$ T cells and $CD8^+$ T cells. (C) Comparison of surface and intracellular markers between $CD20^{dim}CD4^+$ and $CD20^-CD4^+$ T cells prior to ocrelizumab treatment (n = 24). (D) Comparison of surface and intracellular markers between $CD20^{dim}CD8^+$ and $CD20^-CD8^+$ T cells prior to ocrelizumab treatment (n = 24). Abbreviations: Tcm, central memory T cells; Tem, effector memory T cells; Temra, terminally differentiated effector memory T cells; Tn, naive T cells. Statistical analysis was performed by fitting the repeated measures mixed-effects model with the Geisser-Greenhouse correction (accounting for missing data and correcting the violation of sphericity), followed by Sidak's multiple comparison to compare the differences among all pairs (A, B), or Wilcoxon matched-pairs signed-rank test followed by multiple comparison correction using False Discovery Rate by Benjamini, Krieger and Yekutieli's two-stage step-up method (C, D). *p* value, **p* < 0.05, ***p* < 0.01, ****p* < 0.001, *****p* < 0.0001.

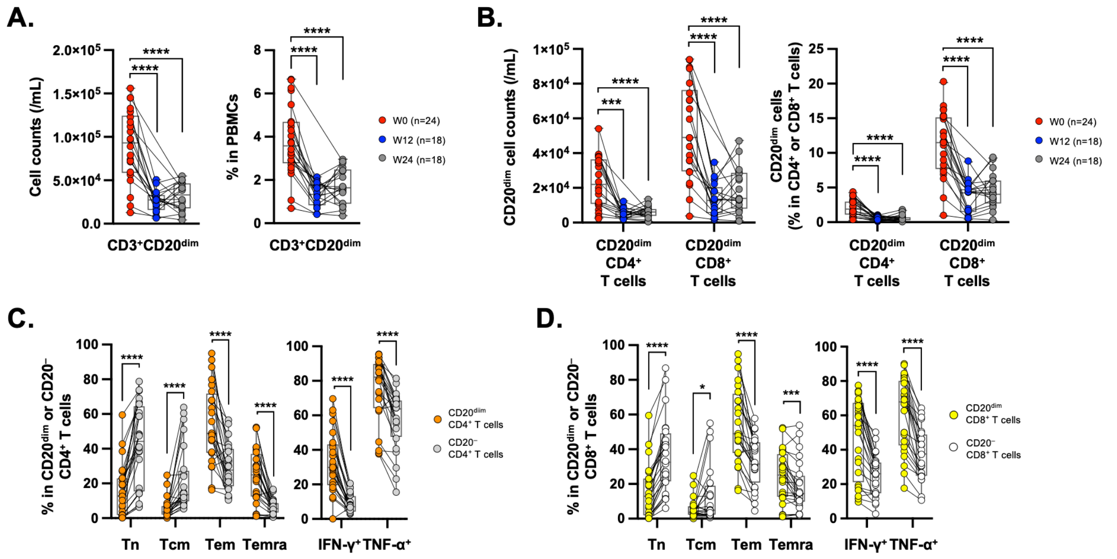

Figure S7. Further characterization of $CD20^{dim}CD4^+$ and $CD20^{dim}CD8^+$ T cells

(A) Comparison of intracellular and surface markers between $CD20^{dim}CD4^+$ and $CD20^–CD4^+$ T cells prior to ocrelizumab treatment (n = 14). (B) Comparison of intracellular and surface markers between $CD20^{dim}CD8^+$ and $CD20^–CD8^+$ T cells prior to ocrelizumab treatment (n = 14). Abbreviations: ALCAM, activated leukocyte-cell adhesion molecule; MCAM, melanoma cell adhesion molecule; NS, not significant. Statistical analysis was performed by Wilcoxon matched-pairs signed-rank test followed by multiple comparison correction using False Discovery Rate by Benjamini, Krieger and Yekutieli's two-stage step-up method. *p* value, **p* < 0.05, ***p* < 0.01, ****p* < 0.001, *****p* < 0.0001.

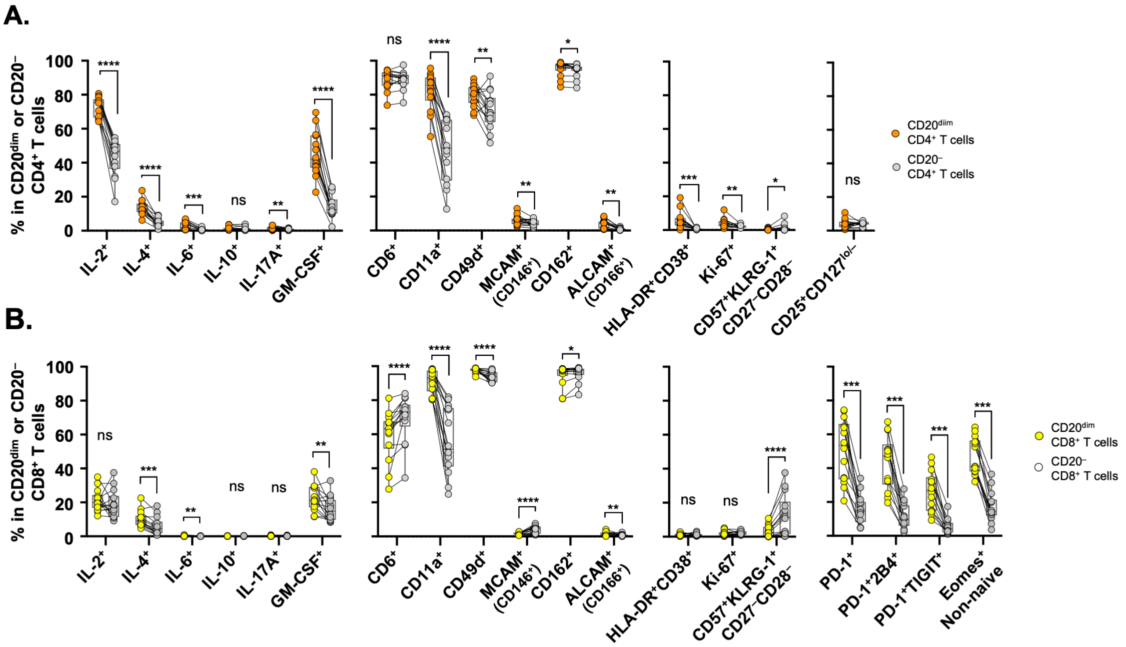

Figure S8. Correlations between treatment-induced changes in CD4+ and CD8+ T-cell counts, and treatment-induced changes in corresponding CD20dim T-cell subsets

Correlations between: (A) treatment-induced changes (Δ) in CD4+ Tem counts and treatment-induced changes CD20dimCD4+ Tem counts; (B) treatment-induced changes in IFN-γ+CD4+ T cell counts and treatment-induced changes in CD20dim IFN-γ+CD4+ T cell counts; (C) treatment-induced changes in TNF-α+CD4+ T cell counts and treatment-induced changes in CD20dimTNF-α+CD4+ T cell counts; (D) treatment-induced changes in CD8+ Tem counts and treatment-induced changes CD20dimCD8+ Tem counts; (E) treatment-induced changes in IFN-γ+CD8+ T cell counts and treatment-induced changes in CD20dimIFN-γ+CD8+ T cell counts; (F) treatment-induced changes in TNF-α+CD8+ T cell counts and treatment-induced changes in CD20dimTNF-α+CD8+ T cell counts. Statistical analysis was performed using Spearman correlation coefficient.

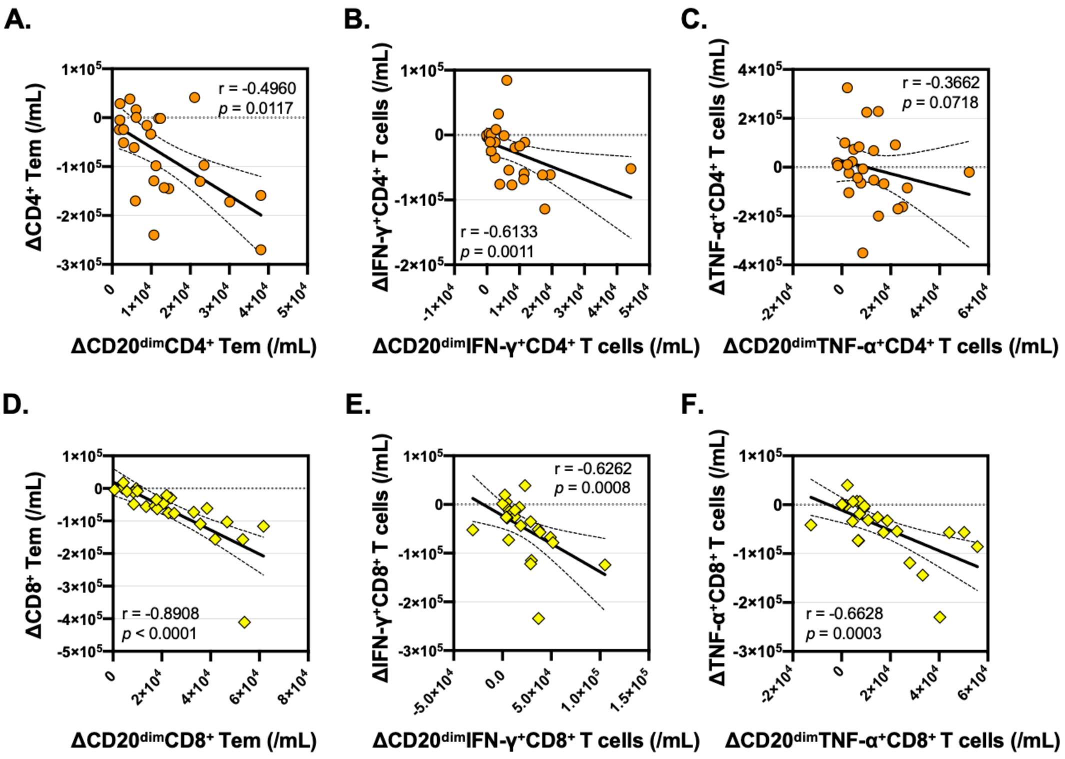

Figure S9. Whole blood cell counts and B-cell phenotype are not associated with disease activity

(A-D) Correlations of the number of Gd-enhancing T1 lesion at baseline with B cell counts (A), CD4⁺ T cell counts (B), CD8⁺ T cell counts (C) and NK cell counts (D). (E-H) Correlations of the number of Gd-enhancing T1 lesion at baseline with frequencies of B-cell subsets, class-switched memory B cells (E), un-class-switched memory B cells (F), naive B cells (G) and transitional B cells (H). Statistical analysis was performed by Spearman correlation coefficient.

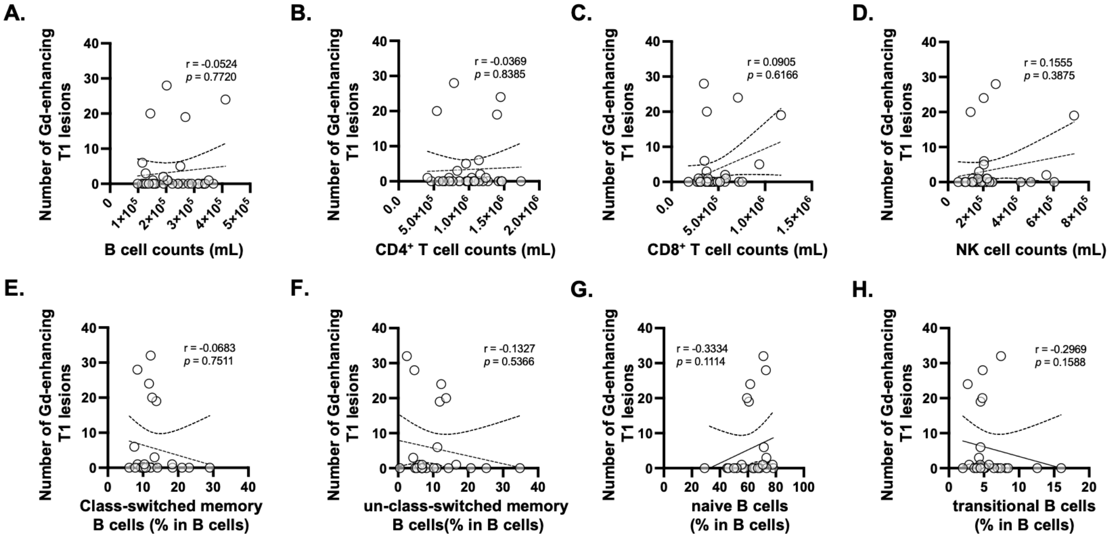

Figure S10. Correlation of B cell recovery with blood sampling time point

Correlation between the frequency of B cells in PBMCs and blood sampling time point (weeks after the first infusion of ocrelizumab) in the combined data of the Discovery and Validation cohorts (n = 58). Statistical analysis was performed using Pearson correlation coefficient since the frequency of B cells and blood sampling time points are normally distributed according to Shapiro-Wilk test (frequency of B cells, W 0.70, $p < 0.0001$; Blood sampling time point, W 0.53, $p < 0.0001$).

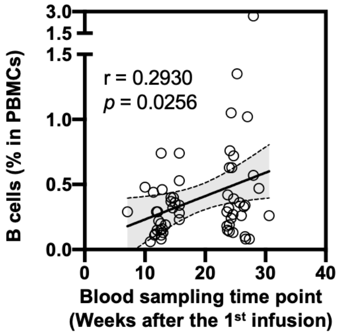

Figure S11. No association between on-treatment new disease activity and subsets of re-emerging B cells and T cells except for CD20^{dim} T cells

(A) Frequencies of B cell, CD4^{+} T cells and CD8^{+} T cells, (B) B cell-subsets, (C) naive/memory CD4^{+} T-cell subsets, (D) naive/memory CD8^{+} T-cell subset, (E) IFN-γ or TNF-α-producing CD4^{+} T cells, (F) IFN-γ or TNF-α-producing CD8^{+} T cells at baseline, at week 12 and at week 24 after ocrelizumab treatment initiation, were compared between patients who did (n = 6, red symbols) or did not (n = 18, blue symbols) experience new disease activity beyond 12 weeks of ocrelizumab treatment. Statistical analysis was performed using multiple Mann-Whitney test. Abbreviations: CSM, class-switched memory B cells, nB, naive B cells; ns, not significant; nT, naive T cells; tB, transitional B cells; Tcm, central memory T cells; Tem. effector memory T cells; Temra, terminally differentiated effector memory T cells; USM, unswitched memory B cells.

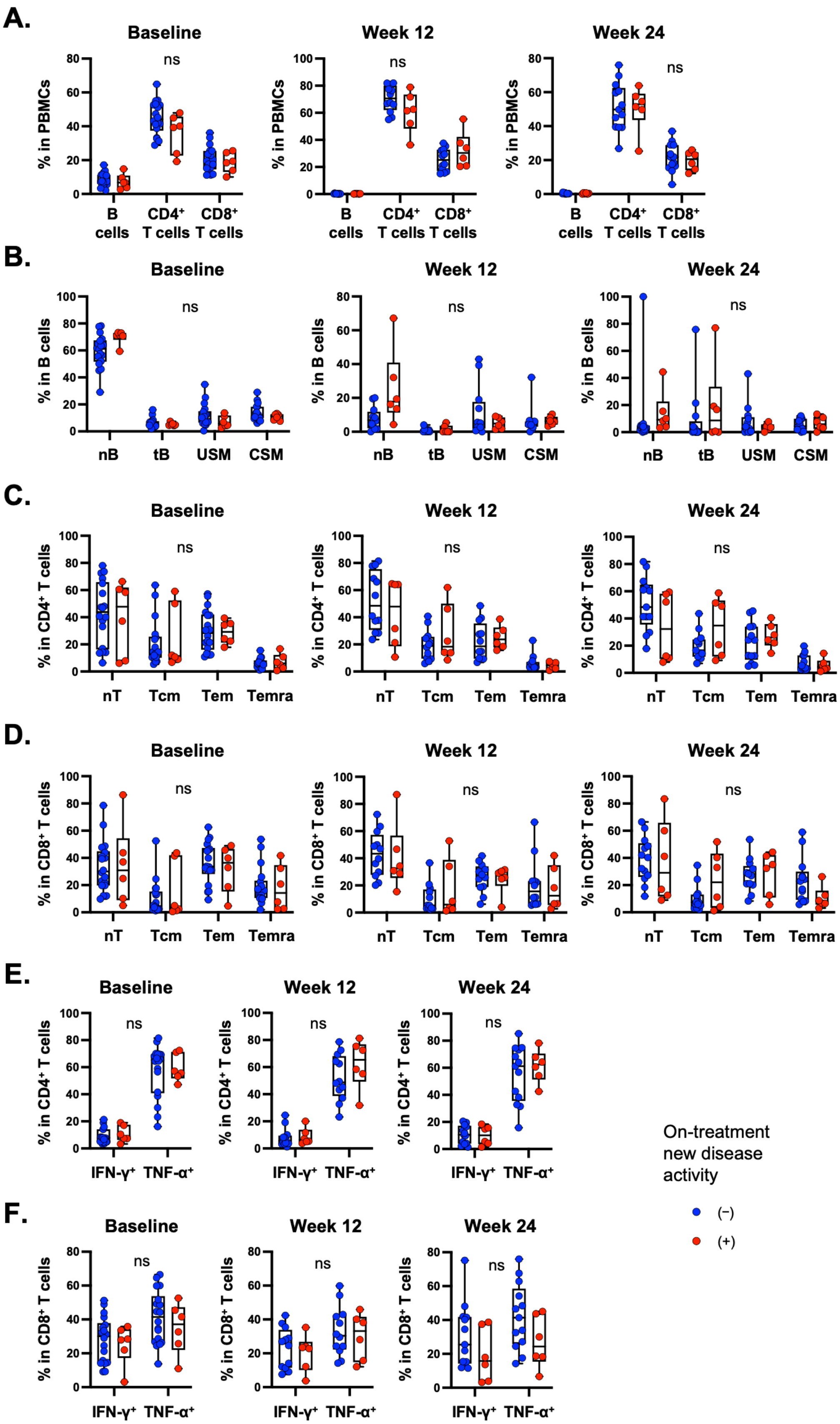
A.
Baseline
Week 12
Week 24
ns
% in PBMCs
B cells
CD4+ T cells
CD8+ T cells
B.
% in B cells
nB
tB
USM
CSM
C.
% in CD4+ T cells
nT
Tcm
Tem
Temra
D.
% in CD8+ T cells
E.
IFN-γ+
TNF-α+
F.
On-treatment new disease activity
(−)
(+)

Table S1. Discovery Cohort: detailed demographics and immune assays performed

Patient ID	Age	Gender	Race	Clinical diagnosis	EDSS	Disease duration (years)†	Duration since last relapse (months)	Prior DMT history	Assays
MS-01	40	F	Black/AA	RRMS	2.5	7	3	None	B cells, T cells
MS-02	40	M	White	RRMS	2.0	0	4	None	B cells, T cells
MS-03	59	M	White	RRMS	3.5	1	4	None	B cells, T cells
MS-04	42	M	White	RRMS	2.0	18	24	None	B cells, T cells
MS-05	24	M	Other	RRMS	2.0	1	8	None	B cells, T cells
MS-06	44	F	Black/AA	RRMS	1.0	2	7	None	B cells, T cells
MS-07	48	M	White	RRMS	1.5	0	5	None	B cells, T cells
MS-08	38	M	White	RRMS	2.0	0	5	None	B cells
MS-09	46	F	White	RRMS	2.5	8	2	None	B cells
MS-10	33	F	Black/AA	RRMS	0.0	2	1	None	B cells
MS-11	25	M	Unknown	RRMS	0.0	3	24	None	B cells
MS-12	54	F	White	RRMS	3.5	2	19	None	B cells
MS-13	45	F	White	RRMS	2.0	5	N/A	None	T cells
MS-14	41	M	Black/AA	SPMS	3.0	8	36	None	B cells, T cells
MS-15	70	F	Black/AA	PPMS	6.5	13	N/A	None	B cells, T cells
MS-16	66	F	White	PPMS	3.5	5	N/A	None	B cells, T cells
MS-17	50	M	Other	PPMS	4.5	1	N/A	None	B cells, T cells
MS-18	65	F	White	PPMS	2.5	9	N/A	None	B cells, T cells
MS-19	60	F	White	PPMS	2.5	3	N/A	None	B cells
MS-20	71	F	Black/AA	PPMS	6.5	18	N/A	None	B cells
MS-21	60	F	Black/AA	PPMS	6.5	3	N/A	None	B cells
MS-22	51	M	White	PPMS	1.0	7	N/A	None	B cells
MS-23	37	M	White	PPMS	2.0	5	N/A	None	T cells

† Disease duration since symptom onset. Abbreviations: AA, African American; EDSS, Expanded Disability Status Scale; DMT, disease-modifying therapy; F, female; M, male; N/A, not available; RRMS, relapsing-remitting MS; SPMS. Secondary progressive MS; PPMS, primary progressive MS.

Table S2. Validation Cohort: detailed demographics and immune assays performed

Patient ID	Age	Gender	Race	Clinical diagnosis	EDSS	Disease duration (years)†	Duration since Last MS Relapse Onset (days)	Number of Gd enhancing lesions at baseline	Prior DMT history	DMT washout duration (days)	Whole blood analysis	PBMC analysis
MS-01	46	M	White	RRMS	2	7.56	N/A	0	GA	13	B, W12, W24	B, W12, W24
MS-02	22	F	White	RRMS	2	2.67	131	24	None		B, W12, W24	B, W12, W24
MS-03	53	M	White	RRMS	5.5	3.73	204	0	IFNβ-1a im.	13	B, W12, W24	B, W12, W24
MS-04	52	F	White	RRMS	2.5	0.74	106	0	IFNβ-1a im.	105	B, W12, W24	B, W12, W24
MS-05	33	F	White	RRMS	1.5	9.79	88	3	DMF	320	B, W12, W24	B, W12, W24
MS-06	33	F	White	RRMS	1	2.61	141	1	None		B, W12, W24	B, W12, W24
MS-07	33	F	White	RRMS	1.5	0.25	78	0	None		B, W12, W24	B, W12, W24
MS-08	28	M	White	RRMS	2	8.96	195	20	GA	>2172	B, W12, W24	B, W12, W24
MS-09	44	F	White	RRMS	4	8.06	228	0	GA	29	B, W12, W24	B, W12, W24
MS-10	34	F	White	RRMS	1.5	0.35	51	0	None		B, W12, W24	B, W12, W24
MS-11	27	F	Black/AA	RRMS	1	4.55	253	0	DMF	294	B, W12, W24	B, W12, W24
MS-12	39	F	White	RRMS	1.5	6.82	207	0	GA	125	B, W12, W24	B, W12, W24
MS-13	36	M	White	RRMS	2	0.84	307	1	None		B, W12, W24	B, W12, W24
MS-14	45	F	Black/AA	RRMS	0	1.3	N/A	0	None		B, W24	B, W12, W24
MS-15	46	F	White	RRMS	2	25.79	253	32	GA	11	W12, W24	B, W12, W24
MS-16	48	F	White	RRMS	1.5	3.93	159	1	None		W12, W24	B, W12, W24
MS-17	27	M	White	RRMS	4.5	0.12	44	19	None		B, W12, W24	B, W12, W24
MS-18	24	M	White	RRMS	1	0.27	97	6	None		B, W12, W24	B, W12, W24
MS-19	50	F	White	RRMS	2	14.84	127	0	GA	62	B, W12, W24	B
MS-20	26	F	Black/AA	RRMS	3	1.03	376	28	None		B, W12, W24	B
MS-21	29	F	White	RRMS	2	10.5	171	0	None		B, W12, W24	B
MS-22	22	M	White	RRMS	2	1.4	146	0	None		B, W12, W24	B
MS-23	28	M	White	RRMS	3	0.59	215	0	None		B, W12, W24	B
MS-24	45	F	Black/AA	RRMS	3	8.48	28	0	IFNβ-1a im.	1268	B, W24	B
MS-25	23	M	White	RRMS	1	0.61	110	0	None		B, W12, W24	
MS-26	53	F	White	RRMS	4	3.13	137	0	None		B, W12, W24	
MS-27	37	M	White	RRMS	0	7.38	380	0	IFNβ-1a sc.	3	B, W12, W24	
MS-28	46	F	White	RRMS	1.5	19.17	303	0	GA	790	B, W12, W24	
MS-29	26	F	Asian	RRMS	1	10.58	483	1	None		B, W12, W24	
MS-30	40	F	White	RRMS	3	2.78	212	0	Fingolimod	488	B, W12, W24	
MS-31	55	M	White	RRMS	2.5	38.76	268	1	IFNβ-1a sc.	89	B, W12, W24	
MS-32	28	M	Black/AA	RRMS	3	1.85	N/A	5	None		B, W12, W24	
MS-33	35	M	White	RRMS	2	0.63	213	2	None		B, W12, W24	
MS-34	47	F	White	RRMS	2	27.86	94	0	DMF	338	B, W12, W24	
MS-35	47	M	White	RRMS	1.5	0.7	N/A	0	None		B, W12, W24	

†Disease duration since symptom onset. Abbreviations: AA, African American; B, baseline; DMF, dimethyl fumarate; DMT, disease-modifying therapy; EDSS, Expanded Disability Status Scale; GA, glatiramer acetate; IFN, interferon; im., intramuscular injection; sc., subcutaneous injection; None, no prior DMT exposure; MS, multiple sclerosis; N/A, not available; RRMS, relapsing-remitting MS; W12, week 12; W24, week 24.

Table S3. Baseline characteristics of MS patients who did or did not develop new disease activity

	New disease activity after W12 n = 6			No new disease activity after W12 n = 29			*p* value
	Mean	SD		Mean	SD		
Age	32.17	8.11		38.41	10.49		NS
Female gender, n (%)	18 (62.1%)			3 (50.0%)			NS
Disease duration †	7.78	9.8		6.62	8.87		NS
EDSS score	1.92	0.66		2.14	1.25		NS
Body weight (kg)	87.5	13.98		82.17	29.63		0.0665
T1 Gd-enhancing T1 lesion count	15	13.45		1.86	5.57		0.0001
T2 lesion count	88.2	35.96		39.17	30.83		0.0113
Baseline brain volume (mL)	1453.3	78.6	n = 5	1430.55	53.33		NS
Normalized cortical grey matter volume (mL)	587.37	13.53	n = 5	587.82	32.86		NS
Normalized thalamic volume (mL)	15532.16	2604.15	n = 5	16759.28	1987.95		NS
T2 lesion volume (mL)	20.14	20.68	n = 5	8.34	11		0.0497
CSF $CD3^+$ T count (/µL)	9.77	9.75		2.87	3.08	n = 23	0.0113
CSF $CD19^+$ B cell count (/µL)	0.51	0.54		0.1	0.16	n = 23	0.0475
CSF GFAP (pg/mL)	697.67	350.64		759.63	341.76	n = 23	NS
CSF NfH (pg/mL)	652	559.67		748.85	408.96	n = 23	NS
CSF NfL (pg/mL)	2001.37	745.81		1706.9	1707.34	n = 23	0.082

†Disease duration after the symptom onset. Abbreviations: CSF, cerebrospinal fluid; EDSS, Expanded disability status scale; Gd, gadolinium; GFAP, glial fibrillary acidic protein; NfL, neurofilament light chain; NfH, neurofilament heavy chain; NS, not significant. Statistical analysis was performed by Fisher's exact test for nominal data, by unpaired t-test for normally distributed data, or by Mann-Whitney test for non-normally distributed data.

Table S4. Immunophenotyping panels for Discovery Cohort

Fluorochrome	FITC (Alexa Flour 488, BB515)	PerCP-Cy5.5 (BB700, PerCP-eFlour 710)	APC (Alexa Flour 647)	APC-R700 (Alexa Flour 700)	APC-H7 (APC-Fire750)	BV421	Aqua	BV605	BV650	BV711	BV786 (BV785)	PE	PE-CF594 (PE-Dazzle 594, PE-Vio615)	PE-Cy7	BUV395	BUV805
T-cell panel 1 (Naive/Memory, Adhesion molecules)	CD6	CD162	CCR7	CD8		CD49d	Live/Dead		CD11a	CD3	CD45RA	ALCAM (CD166)	MCAM (CD146)	CD20		CD4
T-cell panel 2 (Naive/Memory, activated cells)	HLA-DR	CD57	CCR7	CD8	CD95	CD28	Live/Dead		CD27	CD3	CD45RA	Ki-67	KLRG-1	CD20	CD38	CD4
T-cell panel 3 (Exhausted T cells)	Eomes	TIGIT	CCR7	CD8		T-bet	Live/Dead		PD-1	CD3	CD45RA	CTLA4	2B4	CD20	CD38	CD4
T-cell panel 4 (Regulatory T cells)	Helios	TIGIT	CD39	CD8	CD45RO	CD25	Live/Dead		Ki-67	CD3	HLA-DR	CD127	Foxp3	CD20	CD31	CD4
T-cell panel 5 (Cytokine-producing T cells)	IL-2	IL-6	IL-10	CD8	TNF-α	TCRgd	Live/Dead	CD56	IL-17	CD3	CD45RA	GM-CSF	IL-4	CD20	IFN-γ	CD4
T-cell panel 6 (MAIT and $CCR2^{+}CCR5^{+}$ T cells)	TCR Vα7.2	TCRγδ	CD161	CD8	TNF-α	CCR2	Live/Dead	CD56	IL-17	CD3	CD45RA	GM-CSF	CCR5	CD20	IFN-γ	CD4
B-cell panel 1 (Major B-cell subsets)	CD3/CD14/ CD16	IgD	CD21	CD86	CD19	CD43	Live/Dead		CD27	CD24	HLA-DR	CD80	CD10	CD11c	CD38	CD20
B-cell panel 2 (Co-stimulatory or co-inhibitory molecule-expressing B cells)	CD3/CD14/ CD16	IgD	CD71	TIM-1	CD19	TIGIT	Live/Dead		CD27	CD24	L-selectin	Ki-67	GITR	CD83	CD38	CD20
B-cell panel 3 (Cytokine-producing B cells)	CD3/CD14/ CD16	IgD	IgA	IgM	CD19	GM-CSF	Live/Dead		CD27	CD24	TNF-α	IL-6	IL-10	IgG	CD38	CD20

Table S5. Immunophenotyping panels for Validation Cohort

Fluorochrome	FITC (Alexa Flour 488, BB515)	PerCP-Cy5.5 (BB700, PerCP-eFlour 710)	APC (Alexa Flour 647)	APC-R700 (Alexa Flour 700)	APC-H7 (APC-Fire750)	BV421	Aqua	BV650	BV711	BV786 (BV785)	PE	PE-CF594 (PE-Dazzle 594, PE-Vio615)	PE-Cy7	BUV395	BUV805
T-cell panel 1 (Naive/Memory, Adhesion molecules)	CD6	CD14	CCR7	CD8	MCAM	CD49d	Live/Dead	CD11a	CD3	CD45RA	CD162	JAML	CD20		CD4
T-cell panel 2 (Naive/Memory, activated cells)	Integrin β7	CCR4	CCR7	CD8	CD20	CCR2	Live/Dead	CCR6	CD3	CD45RA	CLA	CCR5	CCR9	CCR3	CD4
T-cell panel 3 (Exhausted T cells)	RANTES	TIGIT	CCR7	CD8	LAG-3	T-bet	Live/Dead	PD-1	CD3	CD45RA	CTLA4	2B4	Eomes	CD38	CD4
T-cell panel 4 (Regulatory T cells)	Helios	TIGIT	CD39	CD8	CD45RO	CD25	Live/Dead	Ki-67	CD3	HLA-DR	CTLA4	Foxp3	CD127	CD31	CD4
T-cell panel 5 (Cytokine-producing T cells)	IL-2		TNF-α	CD8	IL-22	IL-10	Live/Dead	IL-17	CD3	CD45RA	GM-CSF	IL-4	CD20	IFN-γ	CD4
T-cell panel 6 (MAIT and CCR2$^+$CCR5$^+$ T cells)	TCRVα7.2		CCR6	CD8	TNF-α	CCR2	Live/Dead	IL-17	CD3	CD45RA	GM-CSF	CCR5	CD161	IFN-γ	CD4
B-cell panel 1 (Major B cell subsets)	CD3/CD14/ CD16	IgD	CD21	CD86	CD19	CD43	Live/Dead	CD27	CD24	HLA-DR	CD80	CD10	CD11c	CD38	CD20
B-cell panel 2 (Co-stimulatory or co-inhibitory molecule-expressing B cells)	CD3/CD14/ CD16	IgD	CD71	TIM-1	CD19	TIGIT	Live/Dead	CD27	CD24	L-selectin	Ki-67	GITR	CD83	CD38	CD20
B-cell panel 3 (Cytokine-producing B cells)	CD3/CD14/ CD16	IgD	IgA	IgM	CD19	GM-CSF	Live/Dead	CD27	CD24	TNF-α	IL-6	IL-10	IgG	CD38	CD20

Table S6. Reagents list

Fluorochrome	Antigen / product	Clone	Supplier	Catalogue #
FITC	CD14	M5E2	BD Biosciences	555397
FITC	CD16	NKP15	BD Biosciences	347523
FITC	CD3	UCHT1	BD Biosciences	555332
FITC	CD6	M-T605	BD Biosciences	555357
BB515	HLA-DR	G46-6	BD Biosciences	564516
FITC	IL-2	MQ1-17H12	BD Biosciences	554565
Alexa Fluor 488	Helios	22F6	Biolegend	137223
FITC	TCR Vα7.2	3C10	Biolegend	351704
FITC	Eomes	WD1928	ThermoFisher Scientific	11-4877-42
BB700	CD162 (PSGL-1)	KPL-1	BD Biosciences	745768
BB700	IgD	IA6-2	BD Biosciences	566538
PerCP-Cy5.5	TCRγδ	B1	BD Biosciences	564157
PerCP-Cy5.5	CD57	QA17A04	Biolegend	393312
PerCP-eFluor 710	IL-6	MQ2-13A5	ThermoFisher Scientific	46-7069-42
PerCP-eFluor 710	TIGIT	MBSA43	ThermoFisher Scientific	46-9500-42
Alexa Fluor 647	CD197 (CCR7)	150503	BD Biosciences	560816
APC	CD21	B-ly4	BD Biosciences	559867
APC	CD39	T66	BD Biosciences	560239
APC	CD86	2331 (FUN-1)	BD Biosciences	565149
APC	IL-10	JES3-19F1	BD Biosciences	554707
APC	CD161	REA631	Miltenyi Biotec	130-113-595
APC	IgA	IS11-8E10	Miltenyi Biotec	130-113-472
APC	CD71	OKT9	ThermoFisher Scientific	17-0719-42
APC-R700	CD8	RPA-T8	BD Biosciences	565165
Alexa Fluor 700	IgM	MHM-88	Biolegend	314538
Alexa Fluor 700	IgM	MHM-88	Biolegend	314538
APC-H7	CD45RO	UCHL1	BD Biosciences	561137
APC-Fire750	CD19	SJ25C1	Biolegend	363030
APC-Fire750	CD19	SJ25C1	Biolegend	363030
APC-Fire750	CD95	DX2	Biolegend	305638
APC-Cy7	TNF-α	Mab11	Biolegend	502944
BV421	CD25	2A3	BD Biosciences	564033
BV421	CD28	CD28.2	BD Biosciences	562613
BV421	CD43	1G10	BD Biosciences	562916
BV421	GM-CSF	BVD2-21C11	BD Biosciences	562930
BV421	IgG	G18-145	BD Biosciences	562581
BV421	TIGIT	741182	BD Biosciences	747844
BV421	CD192 (CCR2)	K036C2	Biolegend	357210
BV421	CD49d	9F10	Biolegend	304322
BV421	T-bet	4B10	Biolegend	644832
BV421	IL-10	JES3-9D7	BD Biosciences	564053
BV421	TCRγδ	B1	Biolegend	331211
Aqua	Live/Dead	Not applicable	ThermoFisher Scientific	L34966
BV605	CD45	HI30	Biolegend	304042
BV605	CD56	HCD56	Biolegend	318334
BV650	CD11a	HI111	BD Biosciences	563934
BV650	CD27	M-T271	BD Biosciences	564894
BV650	CD27	M-T271	BD Biosciences	564894
BV650	IL-17A	N49-653	BD Biosciences	563746
BV650	Ki-67	B56	BD Biosciences	563757
BV650	CD279 (PD-1)	EH12.2H7	Biolegend	329950
BV711	CD24	ML5	BD Biosciences	563401
BV711	CD3	UCHT1	BD Biosciences	563725
BV786	CD45RA	HI100	BD Biosciences	563870
BV786	CD62L (L-selectin)	SK11	BD Biosciences	565311
BV786	HLA-DR	G46-6	BD Biosciences	564041
BV785	TNF-α	Mab11	Biolegend	502948
PE	CD127	HIL-7R-M21	BD Biosciences	557938
PE	CD166 (ALCAM)	3A6	BD Biosciences	559263
PE	CD80	L307.4	BD Biosciences	557227
PE	GM-CSF	BVD2-21C11	BD Biosciences	554507
PE	IL-6	MQ2-13A5	BD Biosciences	554545
PE	CD152 (CTLA-4)	BNI3	Biolegend	369604
PE	Ki-67	REA183	Miltenyi Biotec	130-100-289
PE-CF594	CD10	HI10a	BD Biosciences	562396
PE-CF594	CD146 (MCAM)	P1H12	BD Biosciences	564327
PE-CF594	CD195 (CCR5)	2D7/CCR5	BD Biosciences	562456
PE-CF594	IL-10	JES3-19F1	BD Biosciences	562400
PE-Dazzle 594	CD244 (2B4)	C1.7	Biolegend	329522
PE-Dazzle 594	IL-4	MP4-25D2	Biolegend	500832
PE-Dazzle 594	KLRG-1	SA231A2	Biolegend	367710
PE-Vio615	CD357 (GITR)	REA1007	Miltenyi Biotec	130-116-842
PE-eFlour 610	FOXP3	PCH101	Thermo-Fisher	61-4776-62
PE-Cy7	CD11c	B-ly6	BD Biosciences	561356
PE-Cy7	CD83	HB15e	BD Biosciences	561132
PE-Cy7	IgG	G18-145	BD Biosciences	561298
PE-Cy7	CD20	2H7	Biolegend	302312
BUV395	CD31	WM59	BD Biosciences	565290
BUV395	CD38	HB7	BD Biosciences	563811
BUV395	IFN-γ	B27	BD Biosciences	563563
BUV805	CD20	2H7	BD Biosciences	564917
BUV805	CD4	SK3	BD Biosciences	564910
Trustain FcX (Fc receptor blocker)			Biolegend	422302
Brilliant Staining Buffer			BD Biosciences	659611
Ficoll-Paque Plus			GE Healthcare	17-1440-03
X-vivo10			Lonza	04-743Q
Human Serum AB			Sigma Aldrich	H4522
Ionomycin			Sigma Aldrich	I9657-1MG
phorbol 12-myristate 13-acetate (PMA)			Sigma Aldrich	P1585-1MG
DNaseI			Stemcell	7900
Cytofix/Cytoperm/GoldiStop			BD Biosciences	554715
eBioscience™ Foxp3 / Transcription Factor Staining Buffer Set			ThermoFisher Scientific	00-5523-00
UltraComp eBeads™ Compensation Beads			ThermoFisher Scientific	01-2222-42
